# First Steps toward the Design of Peptides that Influence the Intracellular Palmitoylation Machinery

**DOI:** 10.1002/cbic.202500218

**Published:** 2025-04-23

**Authors:** Katharina Stillger, Eric Platz‐Baudin, Florian Friedland, Melina Ruppel, Coco‐Louisa Sticker, Anne Bodenhausen, Erik Noetzel, Ines Neundorf

**Affiliations:** ^1^ Department of Chemistry and Biochemistry Institute for Biochemistry University of Cologne Zülpicher Straße 47a 50674 Cologne Germany; ^2^ Institute of Biological Information Processing 2: Mechanobiology Forschungszentrum Jülich Wilhelm‐Johnen‐Straße 52428 Jülich Germany

**Keywords:** cysteine palmitoylations, epidermal growth factor receptors, peptides, protein modifications, ZDHHC enzymes

## Abstract

Protein S‐palmitoylation is a reversible posttranslational modification transferring the 16‐carbon fatty acid palmitate to cysteines. It plays a critical role in many cellular processes by influencing protein function, localization, stability, and protein–protein interactions and has a significant impact on various physiological and pathological conditions. This emphasizes the need to develop new technologies to study and treat diseases associated with aberrant palmitoylation. To address these challenges, cell‐permeable peptides containing an Asp–His–His–Cys (DHHC) palmitoylation motif are presented aiming to affect intracellular protein S‐palmitoylation. A small library of peptides is generated and screened for cellular uptake and cell compatibility. Interestingly, the newly designed peptides internalize to high extent into different cell lines and human breast cell spheroids dependent on their palmitoylation motif. In addition, out of this screen, DC‐2 is identified as very potent and this peptide is investigated in more detail concerning its impact on palmitoylated proteins that are connected to cancer progression. These initial explorations highlight that DC‐2 affected the localization of HRas and altered S‐palmitoylation‐related signaling cascades of epidermal growth factor receptor. These findings suggest a peptide‐driven impact on proteins having palmitoylation sites and highlight cell‐permeable DHHC peptides as a potential tool to be further evolved in the context of palmitoylation and cancer.

## Graphical abstract

Herein, DC peptides consisting of the cell‐penetrating peptide sC18* and a Asp–His–His–Cys (DHHC) motif derived from human ZDHHC palmitoyltransferase are highlighted. DC peptides demonstrate high cellular internalization into various cellular models, exert cytotoxic effects against cancerous cells, and alter the function and localization of known palmitoylation substrate proteins.

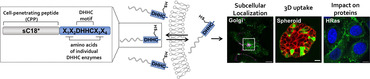

## 1 Introduction

Posttranslational modifications (PTMs) are extremely diverse and enrich the complexity of the proteome. S‐palmitoylation of proteins is a common PTM among proteins, with about 10–20% of human proteins suspected to be palmitoylated.^[^ ^1^, ^2^ ^]^ It is a reversible PTM in which cysteine residues of substrate proteins become modified with the 16‐carbon fatty acid palmitate. This lipid tail primarily enables proteins to anchor to membranes but also plays a role in protein trafficking, stability, and activity.^[^ ^3^ ^]^ Inside mammalian cells, protein S‐palmitoylation is catalyzed by the 23 members of the family of palmitoyl‐S‐acyltransferases (PATs, also called ZDHHC enzymes), all containing a catalytic DHHC (Asp–His–His–Cys) motif. ZDHHC enzymes are integral membrane proteins and predominantly reside at the Golgi or endoplasmatic reticulum (ER), but some are also found at the plasma membrane.^[^ ^4^, ^5^ ^]^ Usually, S‐palmitoylation occurs in a two‐step mechanism, with the first step of “auto‐palmitoylation” being the reaction of the cysteine in the DHHC motif with palmitoyl‐Coenzyme A (CoA) to form the PAT‐acyl intermediate. Next, the palmitate moiety is transferred to a cysteine of a substrate protein.^[^ ^6^, ^7^, ^8^ ^]^ However, protein S‐palmitoylation is a reversible PTM and protein depalmitoylation is catalyzed by acyl protein thioesterases APT1 and APT2, palmitoyl‐protein thioesterase 1 PPT1 and PPT2, and α/β hydrolase domain ABHD10 or ABHD17A–C.^[^ ^9^, ^10^, ^11^, ^12^, ^13^, ^14^, ^15^ ^]^ In fact, S‐palmitoylation is a highly dynamic process in which proteins switch between palmitoylated and depalmitoylated states within seconds to hours.^[^ ^16^, ^17^ ^]^

Abnormalities in protein palmitoylation have been associated with many diseases, including immunodeficiency conditions, neurological disorders, or cancer.^[^ ^18^ ^]^ Especially in cancer, it is known that many tumor suppressors and oncogenes are palmitoylated and that this palmitoylation plays an important role in the formation and progression of tumors. Prominent examples are the small GTPases HRas, NRas, KRas4A, the receptor tyrosine kinases epidermal growth factor receptor (EGFR), c‐Met, p53, SCRIB proteins, and many more.^[^ ^19^, ^20^, ^21^, ^22^, ^23^, ^24^ ^]^ In this respect, also PATs are suggested to act as tumor suppressors or oncogenic proteins. For example, ZDHHC14 is linked to prostate cancer and testicular germ cell tumors where it showed only a low expression and has been identified to act as a tumor suppressor.^[^ ^25^ ^]^ In contrast, ZDHHC17 is hypothesized to act as an oncogene as ZDHHC17 mRNA is overexpressed in many cancers, including breast, prostate, stomach, lung, and colon cancer.^[^ ^26^, ^27^ ^]^

To get a deeper insight into the mechanisms that control S‐palmitoylation dynamics, there is a crucial need for suitable biochemical tools. One of the most used protein S‐palmitoylation inhibitors to date is 2‐bromopalmitate (2‐BP), which is proposed to irreversibly modify the catalytic cysteine within the DHHC motif of PATs.^[^ ^28^, ^29^ ^]^ Unfortunately, 2‐BP is toxic and exhibits several off‐target effects. In fact, 2‐BP directly inhibits proteins involved in lipid metabolism, such as fatty acyl CoA ligase or glycerol‐3‐phosphate acyltransferase, or disturbs other lipid‐related PTMs. Moreover, 2‐BP acts as a deacylation inhibitor by inhibiting the depalmitoylating enzymes APT1 and APT2.^[^ ^29^, ^30^, ^31^, ^32^ ^]^ Recently, the compound cyano‐myracrylamide (CMA), which covalently inhibits ZDHHC enzymes, was reported.^[^ ^33^ ^]^ In comparison to 2‐BP, CMA is significantly less toxic, displays similar potency as 2‐BP, and has no effects on APT1 and APT2. However, for a clickable analogue of CMA, off‐target effects were detected, demanding for improvements and probably new strategies to identify tools to study and influence intracellular S‐palmitoylation.

**Figure 1:**
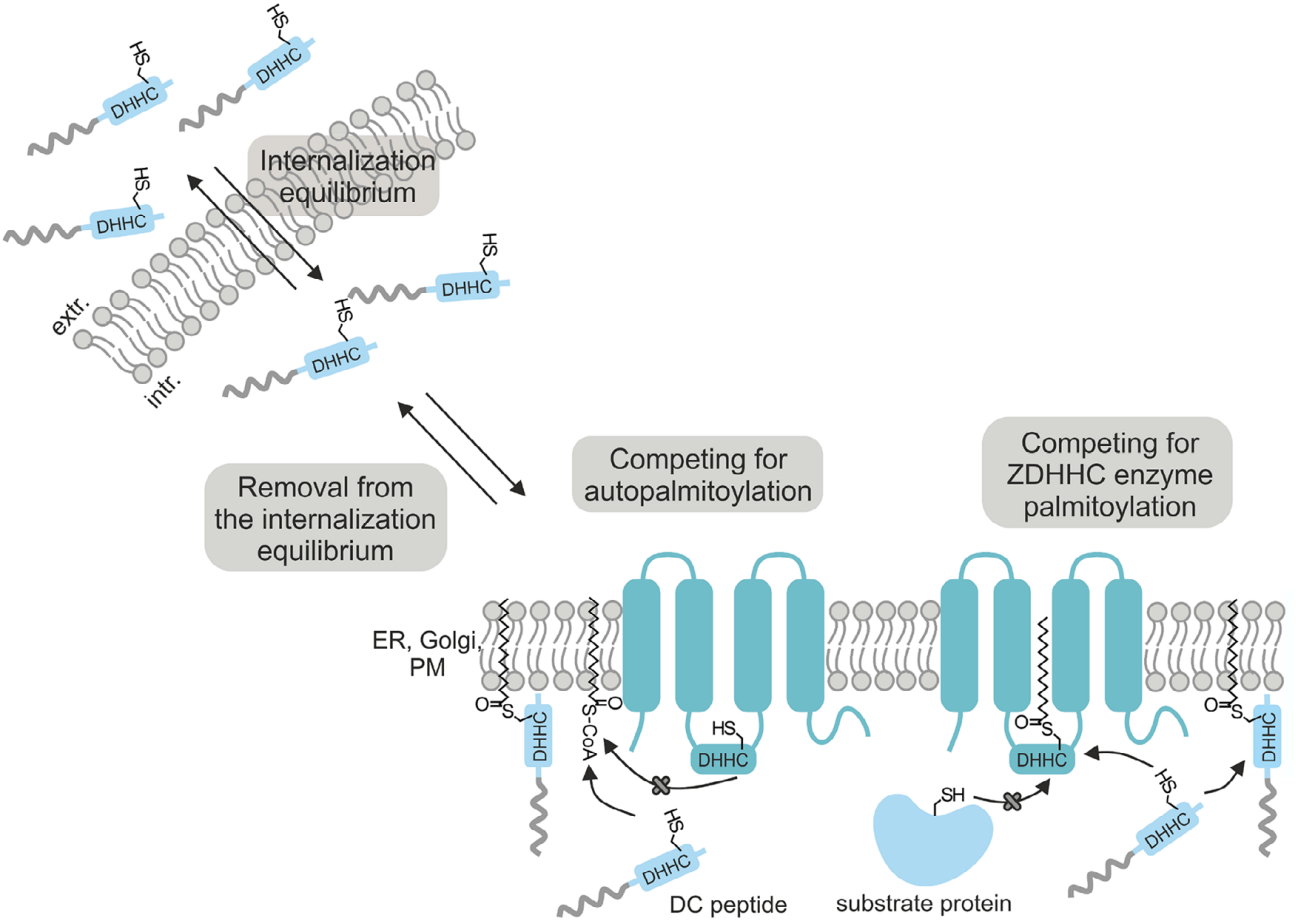
Schematic illustration of internalization and possible intracellular modification of DC peptides. After cellular uptake, we suggest an intracellular palmitoylation of DC peptides that would likely induce the removal of the peptides from an internalization equilibrium at the plasma membrane and triggering intracellular enrichment (extr: extracellular, intr.: intracellular).

Previously, we have successfully designed so‐called CaaX peptides that comprised the cell‐penetrating peptide (CPP) sC18* and a CaaX motif derived from the small GTPases Ras.^[^ ^34^ ^]^ We demonstrated that these peptides display significant biological activity dependent on the presence of the CaaX‐motif and, for instance, altered downstream signaling cascades of KRas. Inspired by these findings, we adapted our previous strategy to possibly target and affect protein S‐palmitoylation (**Figure** **1**). Therefore, we generated a library of so‐called DC peptides in which each peptide included the CPP sC18* fused to respective DHHC motifs derived from the 23 human ZDHHC enzymes. We hypothesized that these peptides would be directly removed from an internalization equilibrium and would likely be further processed via DHHC enzymes (Figure 1). After an initial screening for cellular uptake and cytotoxicity, we analyzed the intracellular fate and activity of the most interesting candidate, namely peptide DC‐2, in more detail. Our results let conclude that DC‐2 treatment in HeLa cells and SW480 colon carcinoma cells might have impacted the localization and functionality of different palmitoylation substrates like HRas and ERK1/2, and likely sensitized EGFR to epidermal growth factor (EGF) stimulation.

## 2 Results and Discussion

### 2.1 Design of Cell‐Permeable DHHC‐Peptides and Biological Screening of the Library

Since there is no reported consensus sequence for proteins that are palmitoylated, the design of peptides interfering with protein S‐palmitoylation was challenging. However, all 23 ZDHHC enzymes contain at least a conserved DHHC sequence that is known to be palmitoylated, which is why we chose this catalytic site as a possible “palmitoylation motif” (Figure 1 and S1, Supporting Information). In addition, a recently presented first structure of the human ZDHHC enzyme, namely ZDHHC20, revealed an important tryptophan residue (Trp158, located two amino acids near to the DHHC motif) interacting with the palmitate moiety in the acyl‐chain binding grove.^[^ ^28^ ^]^ Therefore, we considered the crucial role of this Trp residue, and, after sequence alignment, we expanded the “palmitoylation motif” with the two most conserved amino acids in these positions at each site, leading to the common sequence “X_1_X_2_DHHCX_3_X_4_” (**Table** **1** and S1, Supporting Information). To enable efficient cellular uptake of the peptides, the cell‐permeable peptide sC18*^[^ ^35^ ^]^ was fused at the N‐terminal of the DHHC motifs resulting in a library of 14 different so‐called DC peptides (Table 1). We also included two control peptides, namely the CPP sC18* itself as well as peptide DA‐1, in which the cysteine of the DHHC motif of DC‐2 was exchanged by an alanine. Additionally, a second batch of all peptides was labeled at the N‐terminus with the fluorophore 5(6)‐carboxyfluorescein (CF) for cellular uptake studies (Figure S2, S3 and Table ST1, ST2, Supporting Information).

**Table 1:** Names, sequences, origin and analytical data of synthesized peptides. MW_(calc.)_: calculated molecular weight, MW_(exp.)_: experimental molecular weight, and Da: Dalton. (grey: sC18*, blue: DHHC motif).

Name	Sequence	Origin	MW_(calc.)_ [Da]	MW_(exp.)_ [Da]	Net charge
DC‐1	GLRKRLRKFRNKGFDHHCKW‐NH_2_	ZDHHC1/11	2582.06	2582.86	+8
DC‐2	GLRKRLRKFRNKKMDHHCPW‐NH_2_	ZDHHC2/3/6/7/15/16/20	2606.15	2606.44	+8
DC‐3	GLRKRLRKFRNKRFDHHCVW‐NH_2_	ZDHHC4	2652.16	2652.77	+8
DC‐4	GLRKRLRKFRNKEFDHHCPW‐NH_2_	ZDHHC5	2623.07	2623.67	+6
DC‐5	GLRKRLRKFRNKDFDHHCPW‐NH_2_	ZDHHC8	2609.04	2609.73	+6
DC‐6	GLRKRLRKFRNKRFDHHCPW‐NH_2_	ZDHHC9/14/18	2650.14	2650.73	+8
DC‐7	GLRKRLRKFRNKRYDHHCPW‐NH_2_	ZDHHC12	2666.14	2666.59	+8
DC‐8	GLRKRLRKFRNKRYDQHCLW‐NH_2_	ZDHHC13	2673.17	2673.66	+8
DC‐9	GLRKRLRKFRNKKFDHHCPW‐NH_2_	ZDHHC17	2622.13	2622.61	+8
DC‐10	GLRKRLRKFRNKDFDHHCKW‐NH_2_	ZDHHC19	2640.10	2640.73	+7
DC‐11	GLRKRLRKFRNKRMDHHCPW‐NH_2_	ZDHHC21	2634.16	2634.65	+8
DC‐12	GLRKRLRKFRNKRHDHHCFF‐NH_2_	ZDHHC22	2651.13	2651.57	+8
DC‐13	GLRKRLRKFRNKRMDHHCVW‐NH_2_	ZDHHC23	2636.18	2636.69	+8
DC‐14	GLRKRLRKFRNKRRDHHCRL‐NH_2_	ZDHHC24	2645.17	2645.83	+10
DA‐1	GLRKRLRKFRNKKMDHHAPW‐NH_2_	DC‐2 Cys ‐> Ala	2574.08	2574.67	+8
sC18*	GLRKRLRKFRNK‐NH_2_	CAP18	1570.94	1571.52	+8

**Figure 2:**
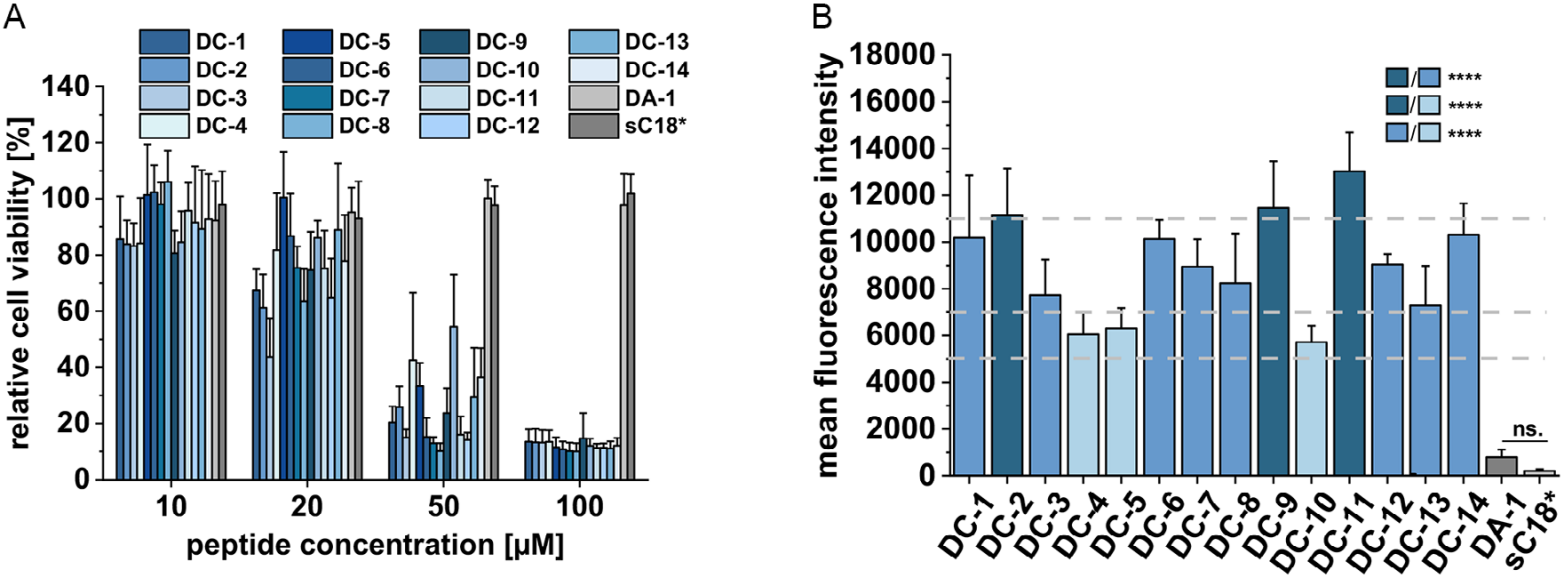
A) Cell viability assay in HeLa cells after 24 h incubation with different peptide concentrations. An untreated control was set to 100% cellular viability (*n* = 3). B) Analysis of peptide uptake into HeLa cells using flow cytometry. Cells were incubated for 30 min with 10 μM CF‐labeled peptides. Peptides were divided into three groups based on their cellular uptake: high (dark blue), medium high (blue), and medium (light blue) (*n* = 3). All values of each group were statistically compared using a one‐way ANOVA (****P ≤ 0.0001, ns.: not significant).

A first physicochemical investigation using circular dichroism (CD) spectroscopy revealed that all peptides including both control peptides were unstructured in buffered solution but formed α‐helical structures when in presence of trifluoroethanol (TFE) (Figure S4, Supporting Information). This agreed with former studies.^[^ ^34^ ^]^ In a next step, we screened all DC peptides and their respective control peptides regarding their cytotoxicity. Therefore, HeLa cells were treated for 24 h with increasing peptide concentrations in the micromolar range. Interestingly, several DC peptides seemed to distinctly impact the cellular viability already at 20 μM and upon treatment with ≥50 μM peptide solutions, all DC peptides were highly cytotoxic (**Figure** **2**A). Compared to this, both control peptides DA‐1 and sC18* were not toxic up to a concentration of 100 μM. For the latter, this is in line with previously published data.^[^ ^34^, ^36^ ^]^ DA‐1 is based on DC‐2, in which only the cysteine has been switched to alanine. Therefore, we assigned the high cytotoxic effect of DC‐2 to the present cysteine within the DHHC motif.

Following, we assessed if the observed cytotoxicity was linked to the internalization ability of the peptides. Thus, HeLa cells were treated for 30 min with 10 μM CF‐labeled peptides and the uptake was measured using flow cytometry. Strikingly, all DC peptides were taken up significantly higher into HeLa cells compared to the control peptides (Figure 2B). This ranged from a 7‐ to 16‐fold increased uptake compared to DA‐1 and 27‐ to 62‐fold increased uptake compared to sC18*, respectively. Since the presence of cysteine instead of alanine within the DHHC motif dramatically increased the uptake, we linked this result to the possible intracellular modification at the respective DHHC motif. Also of note is that the internalization level of DC peptides was varied; therefore, we ordered them into three groups depending on high (dark blue), medium‐high (blue), or medium (light blue) cellular uptake. The relatively lower uptake observed for the medium peptide group might be attributed to the presence of negatively charged Asp and Glu residues in the amino acid sequence (Table 1).^[^ ^37^ ^]^ Three peptides were then chosen for further studies, including DC‐2 as this peptide contained the most conserved DHHC motif among all palmitoyl transferases, DC‐11 since this was the peptide with the highest uptake, and DC‐4, a peptide that internalized to less extent.

### 2.2 Analyzing Cytotoxicity and Cellular Uptake of Selected DC Peptides

**Figure 3:**
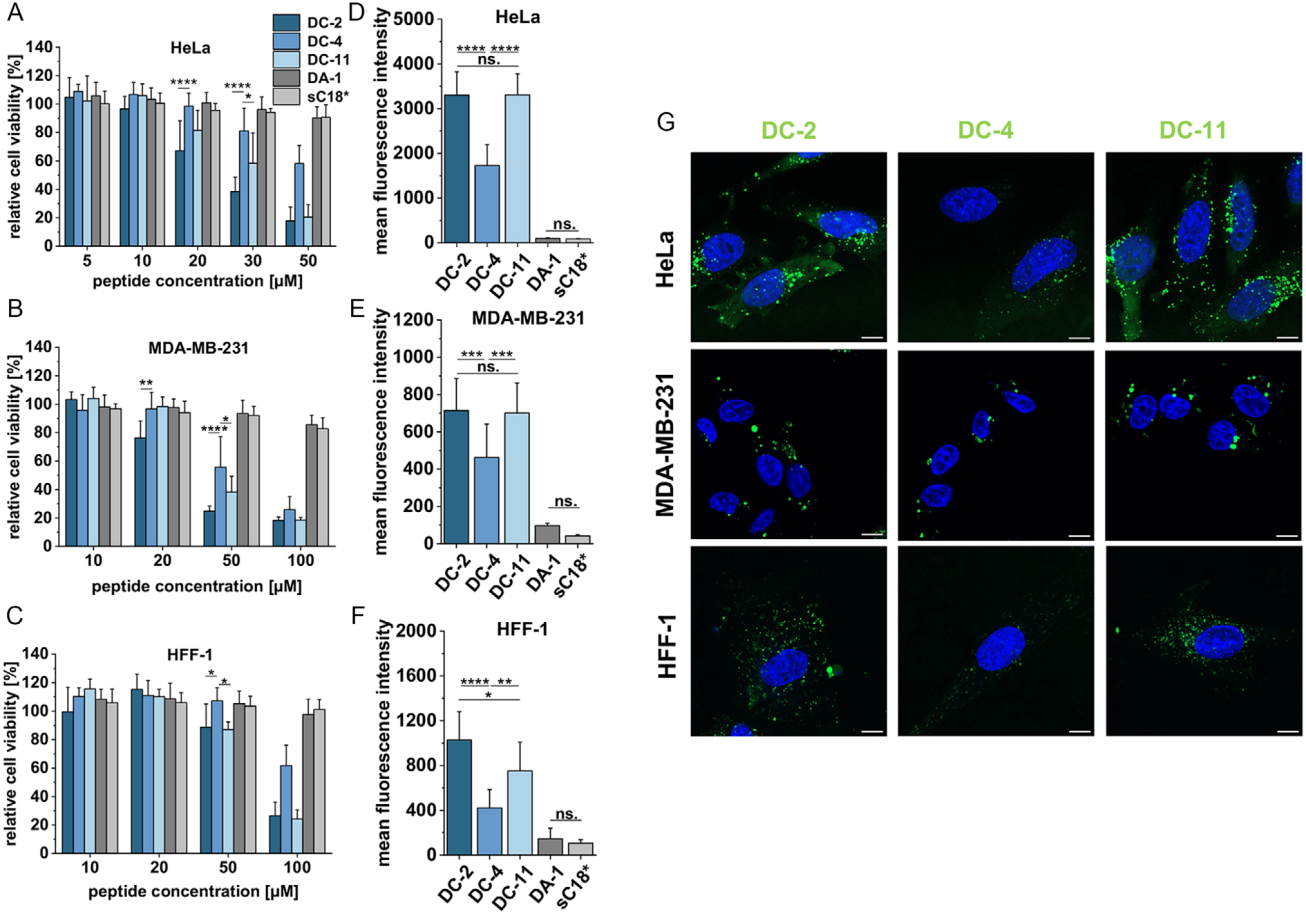
Cell viability assay in A) HeLa cells, B) MDA‐MB‐231, and C) HFF‐1 cells. Cells were incubated for 24 h with different concentrations of the CF‐labeled peptides (*n* = 3). An untreated control was set to 100% cellular viability. Statistical analyses were performed using a two‐way ANOVA (*P ≤ 0.05, **P ≤ 0.01, ***P ≤ 0.001, ****P ≤ 0.0001). Analysis of peptide uptake into D) HeLa, E) MDA‐MB‐231, and F) HFF‐1 cells using flow cytometry. Cells were incubated for 30 min with 5 μM CF‐labeled peptides (*n* ≥ 3). Statistical analyses were performed using a one‐way ANOVA (*P ≤ 0.05, **P ≤ 0.01, ***P ≤ 0.001, ****P ≤ 0.0001, ns: not significant). G) Analysis of peptide uptake into HeLa, MDA‐MB‐231, and HFF‐1 cells using live‐cell fluorescence microscopy. Cells were incubated for 30 min with 5 μM CF‐labeled peptides. Blue: Hoechst 33342 nuclear stain, green: CF‐labeled peptide, and scale bar: 10 μm.

The three selected peptides were then evaluated in more detail concerning their cytotoxicity profiles. In addition to HeLa cells, we included the breast cancer cell line MDA‐MB‐231, as well as noncancerous human foreskin fibroblasts (HFF‐1). After 24 h treatments, both additionally tested control peptides, DA‐1 and sC18*, were not toxic when incubated with all three cell lines, proving again the importance of the present cysteine. Also, we recognized that attachment of the fluorophore did not negatively influence the peptides since they exhibited similar toxicity against HeLa cells compared to their unlabeled variants (**Figure** **3**A). DC‐2 was the most active peptide in all cell lines tested followed by DC‐11 and DC‐4, while HFF‐1 cells were only severely affected after applying peptide solutions of 100 μM (Figure 3C). This let suggest different uptake and activity mechanisms in cancerous cells.

Next, we verified if the observed different activities between cancerous and noncancerous cells were related to the membrane activities of the peptides. For this, we used red blood cells (RBCs), which have a neutrally charged membrane and were, therefore, often taken as model system to study membrane activity.^[^ ^38^ ^]^ Incubating RBCs for 24 h with 100 μM peptide concentrations demonstrated that the hemolysis rate for all three DC peptides ranged between 9% and 16% (Figure S5A, Supporting Information), letting us conclude that they were not hemolytic. Then, we investigated the impact of all three DC peptides on the integrity of the plasma membrane of HeLa cells using a lactate dehydrogenase (LDH) assay. In fact, after treating cells for 1 h with 20 μM of the respective DC peptide, a significant amount of LDH was released, which further increased with higher concentrations, while both control peptides did not lead to any significant LDH release (Figure S5B, Supporting Information). Interestingly, when removing the peptide solution after 1 h to allow the cells to rest for 5 h, only cells treated with 50 μM of the DC peptides released a low extent of LDH, highlighting that cell membranes might recover from the peptide treatment (Figure S5C, Supporting Information).

According to these results, we used lower concentrations to quantify cell internalization of the peptides, e.g. 5 μM peptide solutions, to outline any negative effects on membrane integrity. Like in our first screen in HeLa cells (Figure 2B), DC‐2 and DC‐11 internalized to a higher extent compared to DC‐4 also in the other cell lines tested (Figure 3D–F). The uptake was quite higher in HeLa compared to MDA‐MB‐213 and HFF‐1 cells for all peptides tested. Moreover, we again hypothesized a significant influence of the cysteine and, thus, possible intracellular modification at this residue since the controls were still taken up to a far less extent. This was also the case for DC‐2 and DC‐11 when only 1 μM peptide concentrations were tested (Figure S5D, Supporting Information). The overall weaker uptake of DC‐4 might be explained by its lower net charge as it contains a Glu instead of a Lys (DC‐2) or Arg (DC‐11) within the palmitoylation motif. Both Lys and Arg have been described as crucial amino acids supporting the first contact of CPPs with the negatively charged phospholipids at the outer surface of the cell membrane.^[^ ^39^, ^40^, ^41^ ^]^ All in all, the cytotoxic activity of the peptides likely correlates with their uptake efficiency.

In addition, we inspected the cells by confocal laser scanning microscopy, and, also then, we observed that DC peptides, particularly DC‐2 and DC‐11, internalized significantly higher compared to control peptides (Figure 3G and S5F, Supporting Information). Interestingly, a mainly punctate uptake pattern was detected in all cell lines, which would speak in favor of endocytotic entry pathways. On the other side, DC‐2 and DC‐11 also showed a diffuse cytosolic pattern in HeLa cells, highlighting that these peptides might also translocate via direct uptake pathways, or they might have already escaped the endosomes.

### 2.3 Impact of the DHHC Motif on Biological Activity of DC Peptides

**Figure 4:**
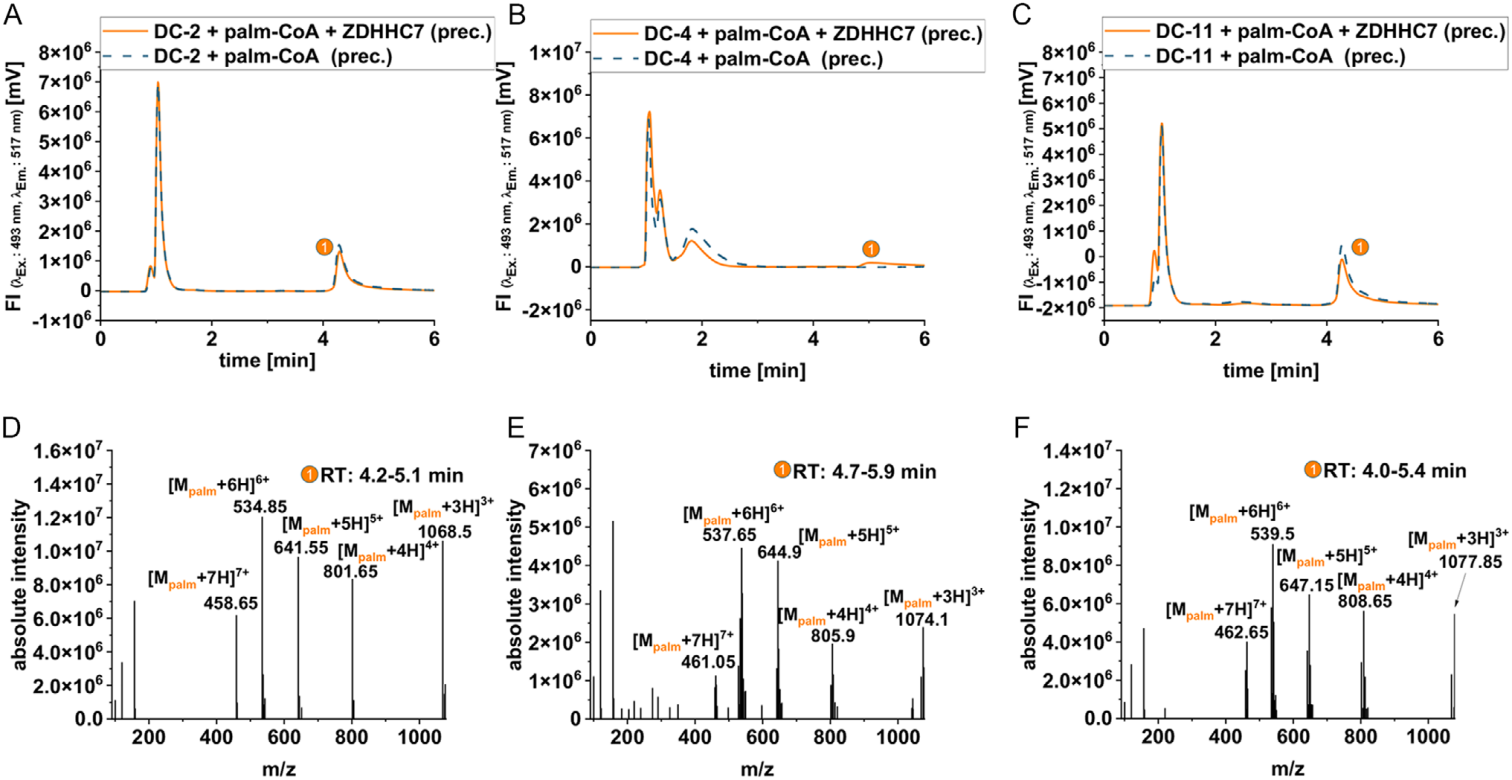
LC‐MS analysis of in vitro palmitoylation reaction of DC peptides with ZDHHC7 and palmitoyl‐CoA. HPLC chromatograms of precipitate of in vitro reaction of A) CF‐DC‐2, B) CF‐DC‐4, and C) CF‐DC‐11. Mass spectrum referring to the indicated peak shows m/z signals that correspond to the quasi‐molecular ions of D) S‐palmitoylated CF‐DC‐2, E) S‐palmitoylated CF‐DC‐4, and F) S‐palmitoylated CF‐DC‐11. The other peaks refer to DMSO and salts eluting from the column or the unmodified DC peptide.

Our results thus far let suggest that DC peptides are likely recognized by the palmitoylation machinery triggering their fast and intensive cellular uptake. Therefore, we tested by liquid chromatography‐mass spectrometry (LC‐MS) analysis whether DC peptides were palmitoylated by the enzyme ZDHHC7. The results revealed that DC‐2 as well as DC‐11 were palmitoylated both in presence and absence of ZDHHC7, indicating auto‐palmitoylation (**Figure** **4**A–F). Compared to this, we found only a minor peak of palmitoylated DC‐4 when in presence of ZDHHC7 and no auto‐palmitoylation (Figure 4B). This would be in line with the formerly observed lower cellular uptake and cytotoxicity of this peptide compared to DC‐2 and DC‐11 suggesting that the peptides were potentially recognized by ZDHHC enzymes.

**Figure 5:**
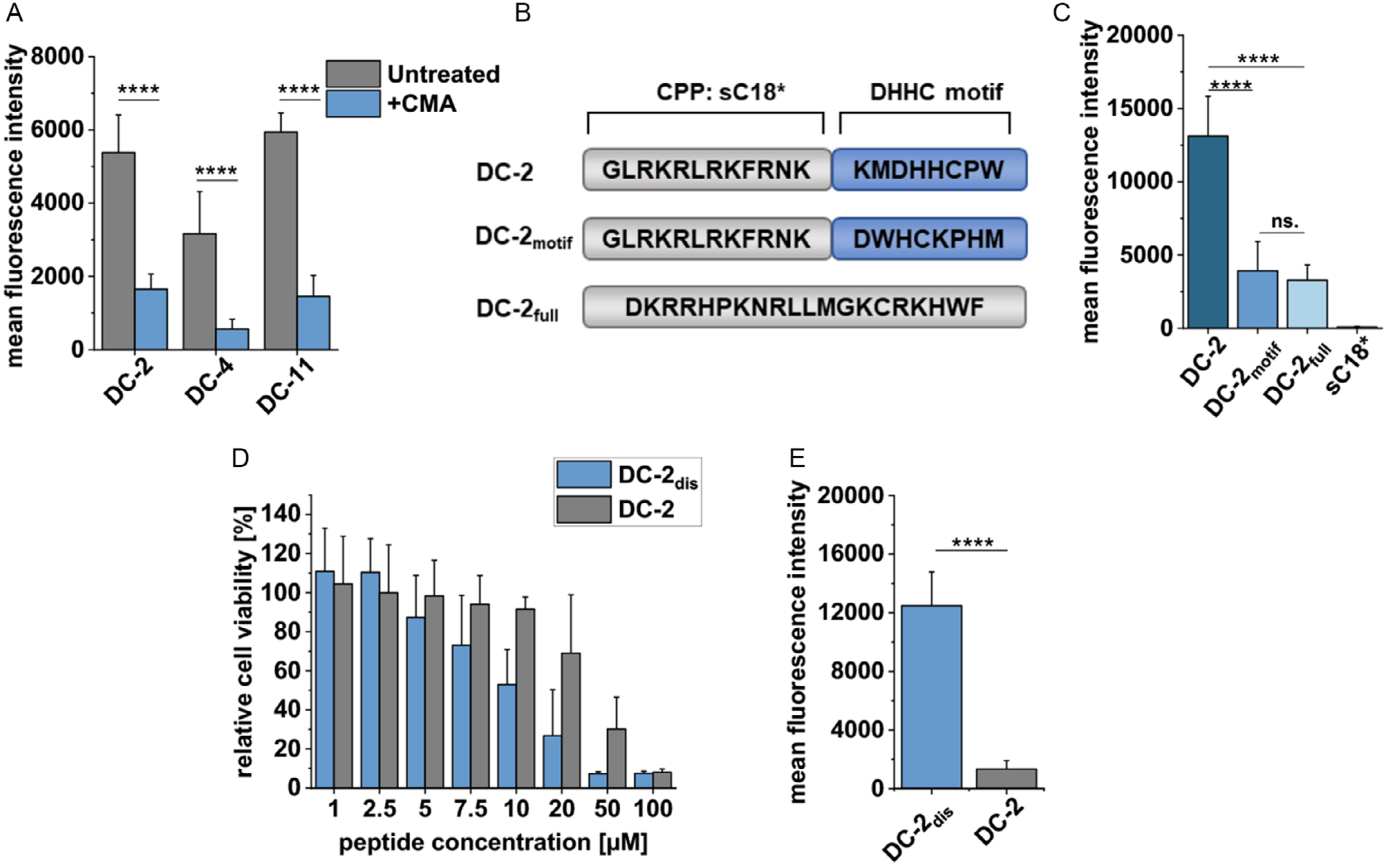
A) Uptake analysis of peptides into HeLa cells using flow cytometry and after inhibition of S‐palmitoylation. Cells were treated for 6 h with 20 μM CMA followed by 5 μM CF‐labeled peptides for 30 min (*n *= 3). Statistical analysis was performed using an unpaired T test (****P ≤ 0.0001). B) Design of scrambled controls. C) Uptake analysis of peptides into HeLa cells using flow cytometry. Cells were incubated for 30 min with 5 μM CF‐labeled peptides (*n *= 3). Statistical analyses were performed using a one‐way ANOVA (****P ≤ 0.0001, ns: not significant). D) HeLa cells were incubated for 24 h with different concentrations of the CF‐DC‐2 and CF‐DC‐2dis (*n *= 3). An untreated control was set to 100% cellular viability. E) HeLa cells were treated with 1 μM CF‐DC‐2 and CF‐DC‐2dis for 30 min to analyze cellular uptake using flow cytometry (*n* = 3). Statistical analysis was performed using an unpaired T test (****P ≤ 0.0001).

To further analyze the impact of the DHHC motif on the cellular uptake, HeLa cells were treated with the recently developed palmitoylation inhibitor CMA.^[^ ^33^ ^]^ Indeed, a drastic and significant decrease in uptake for all three DC‐peptides was detected (**Figure** **5**A). We then generated scrambled controls based on DC‐2, in which either the DHHC motif (DC‐2_motif_) or the whole peptide sequence was scrambled (DC‐2_full_) (Figure 5B and S6, Supporting Information). Interestingly, when analyzing cytotoxicity and cellular uptake of both scrambled peptides DC‐2_motif_ and DC‐2_full_, they exhibited less cytotoxicity compared to DC 2 (Figure S7A, Supporting Information), and they were also significantly less internalized (Figure 5C). CD spectroscopy showed the formation of amphipathic helical structures pointing to similar physicochemical characteristics to the parent DC‐2 peptide (Figure S7C,D, Supporting Information). All in all, we speculate again that the reduced uptake was more likely related to the missing intact DHHC motif.

In the following experiments, we aimed to exclude any other nonspecific effects of the cysteine on the cellular uptake of DC peptides. We chose again DC‐2 for these studies and first analyzed if this peptide would form dimers in the oxidative extracellular environment. It has been recently shown that dimerization can greatly enhance the uptake of CPPs.^[^ ^42^, ^43^ ^]^ Therefore, we synthesized a disulfide variant of DC‐2 (Figure S6, Supporting Information) and studied its internalization and cytotoxicity. As expected, DC‐2_dis_ exhibited enhanced cytotoxicity and was clearly taken up stronger than DC‐2. Thus, we suggested that DC‐2 might not form extracellular dimers (Figure 5D,E). We verified this hypothesis by analyzing the cell culture medium using LC‐MS either before the addition of DC‐2 to cells or after 30 min incubation. Indeed, when DC‐2 was added to normal cell culture medium, we did not observe the disulfide dimer of DC‐2. Surprisingly, at both incubation times, we observed a peptide species with a Δmass = +119 Da (Figure S8A, Supporting Information), which we assigned to an S‐cysteinylation at the cysteine potentially originating from L‐cystine present in the cell culture medium (Figure S8D, Supporting Information). To verify this finding, we performed the same experiment in cell culture medium lacking L‐cystine and then mostly reduced peptide was observed, confirming cysteinylation as the underlying modification (Figure S8A, Supporting Information). This modification has been previously observed also for other cysteine‐containing peptides developed in our group.^[^ ^43^ ^]^ Additionally, we also identified the DC‐2 disulfide dimer and another species with Δmass = +305 Da, potentially representing S‐glutathionylation at the cysteine. However, under normal cell culture conditions, only cysteinylation was observed. Additionally, due to the reductive environment of the cytosol, we expect that the cysteinylated peptide is reduced after cellular uptake by the intracellular glutathione pool.^[^ ^44^, ^45^ ^]^ In a last step, we tested the cellular uptake of DC‐2 in HeLa cells cultured in medium lacking L‐cystine (Figure S8B, Supporting Information). Here, the uptake of the peptide was slightly increased, possibly as a result of the formed DC‐2 disulfide dimers that support more efficient internalization in this case (Figure S8A,B, Supporting Information).

Other nonspecific effects to be excluded were related to thiol‐exchange reactions at the plasma membrane, which were described previously for cysteine‐containing peptides.^[^ ^46^, ^47^, ^48^ ^]^ Therefore, we blocked surface thiols of HeLa cells by using an excess of Ellman's reagent (5,5‐dithio‐bis‐2‐nitrobenzoic acid [DTNB]) followed by addition of the peptide and analysis of cellular uptake (Figure S8C, Supporting Information). While we observed a significant difference for the disulfide variant of DC‐2, highlighting an uptake mechanism dependent on cell surface thiols, no significant difference was observed for the reduced DC‐2.

Based on all these results, we propose that internalization and cytotoxicity of DC peptides are based on the presence of an intact DHHC motif. We hypothesize that they presumably encounter further processing by the palmitoylation machinery after cellular uptake and, therefore, are being removed from the internalization equilibrium, what likely leads to the detected high intracellular accumulation (Figure 1). This potential interference with the intracellular palmitoylation machinery might then provoke additional cytotoxic effects.

### 2.4 DC‐2 Localizes at Multiple Subcellular Destinations and Infiltrates Basement Membrane (BM)‐Covered Spheroids

To get a deeper understanding of the uptake, intracellular fate, and internalization mechanism of DC peptides, we chose DC‐2 as a representative candidate. At first, we analyzed if any receptor‐ or transporter‐dependent uptake would play a role for internalization of this peptide. When HeLa cells were pretreated with 5 μM unlabeled DC‐2, we measured no significant difference in the quantity of peptide uptake (Figure S5E, Supporting Information). Therefore, we concluded receptor‐/transporter‐independent cell internalization.^[^ ^49^ ^]^ Subsequent time‐lapse studies in which HeLa cells were treated for 30 min with 5 μM DC‐2 revealed rapid cellular uptake and the presence of large peptide aggregates at the cellular membrane (Video 1). These aggregates might be assigned to so‐called “nucleation zones”, which were previously described for arginine‐rich peptides.^[^ ^50^ ^]^

**Figure 6:**
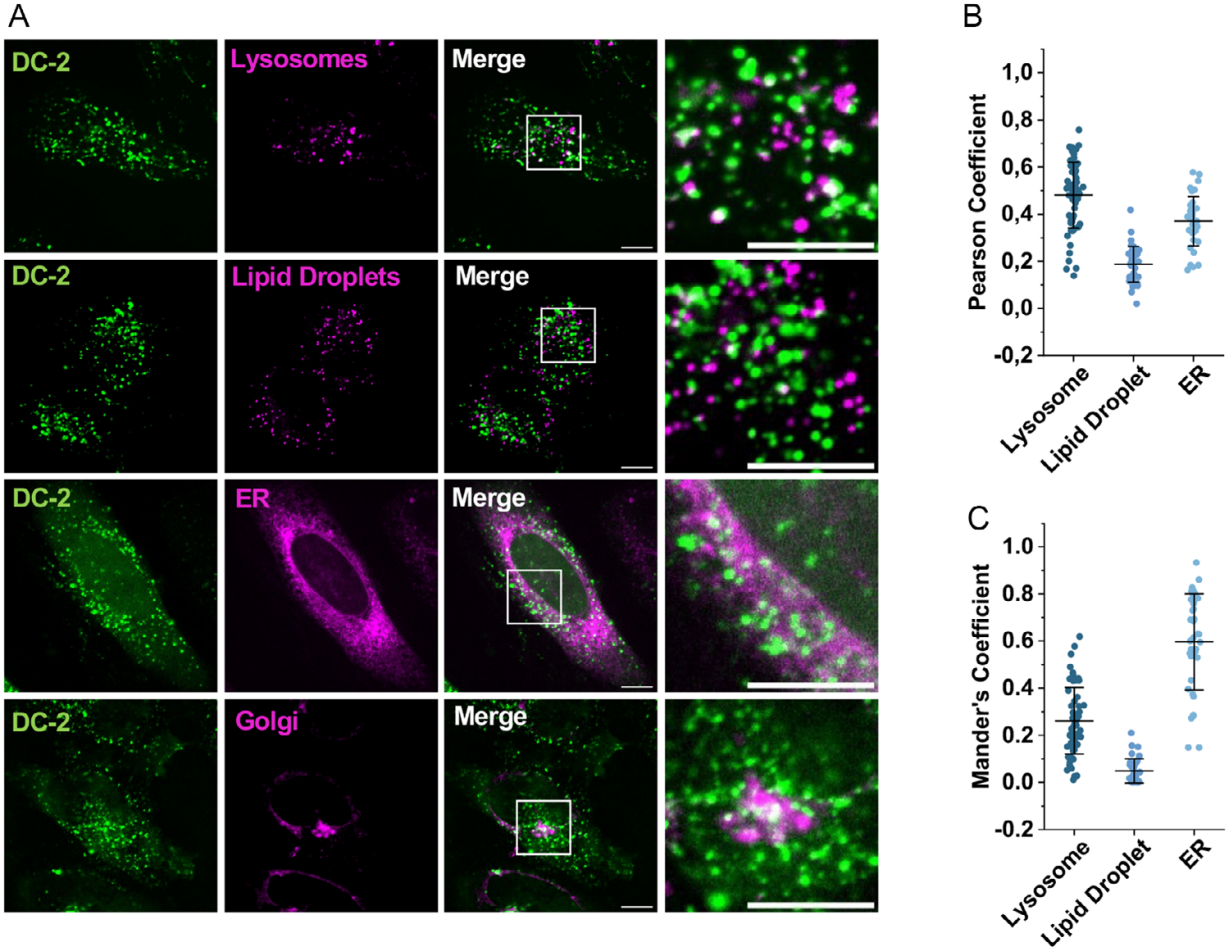
Co‐localization studies of DC‐2 using different stains. A) HeLa cells were incubated for 30 min with 5 μM CF‐DC‐2 and LysoTracker Red DND‐99 (Invitrogen), or a distyryl Bodipy lipid droplet stain synthesized and provided by Engelhardt et al.^[^ ^67^ ^]^ For staining the ER, HeLa cells were treated over night with CellLight ER‐RFP, BacMam 2.0 (Invitrogen) and on the next day, 5 μM CF DC‐2 were added for 2 h. For staining the Golgi, HeLa cells were incubated for 2 h with 5 μM CF‐DC‐2 and for the last 30 min of the incubation time, BODIPY TR ceramide complexed to BSA (Invitrogen) was added. Scale bars: 10 μm. B,C) Pearson and Manders’ coefficients were calculated using the JaCoP analysis tool.^[^ ^68^ ^]^ Data represents mean ± SD of ≥37 cells of *n* = 2. For Golgi analysis, no PCC or MOC were determined as the unedited images contained a too strong background signal.

However, we then performed co‐localization experiments staining lysosomes, lipid droplets, Golgi, and ER (**Figure** **6**). To quantify the degree of co‐localization, we calculated the Pearson correlation coefficient (PCC) and the Mander's overlap coefficient (MOC). After 30 min incubation in HeLa cells, we found that only a fraction of DC‐2 peptides localized in lysosomes (PCC of 0.48 ± 0.14 and MOC of 0.26 ± 0.14) (Figure 6). Since PCC and MOC were near to zero (PCC of 0.19 ± 0.08 and a MOC of 0.05 ± 0.05) when the lipid droplet stain was utilized, a more random co‐localization was supposed in this case. As most ZDHHC enzymes are present at the Golgi or ER, we next analyzed co‐localization of DC‐2 with these organelles. Compared to 30 min, after 2 h incubation, the peptide signal of DC‐2 was more diffuse, but punctate patterns inside cells were also visible. Notably, co‐localization of DC‐2 peptides at both organelles (for ER, we measured PCC of 0.37 ± 0.10 and MOC of 0.60 ± 0.20) was detectable (Figure 6). Interestingly, after incubating DC‐2 for 6 h, the peptide predominantly accumulated on one side of the cell nuclei which likely represented localization at the Golgi (Figure S9, Supporting Information). Altogether, we concluded that DC‐2 peptides might be localized to palmitoylation sites after cellular entry.

**Figure 7:**
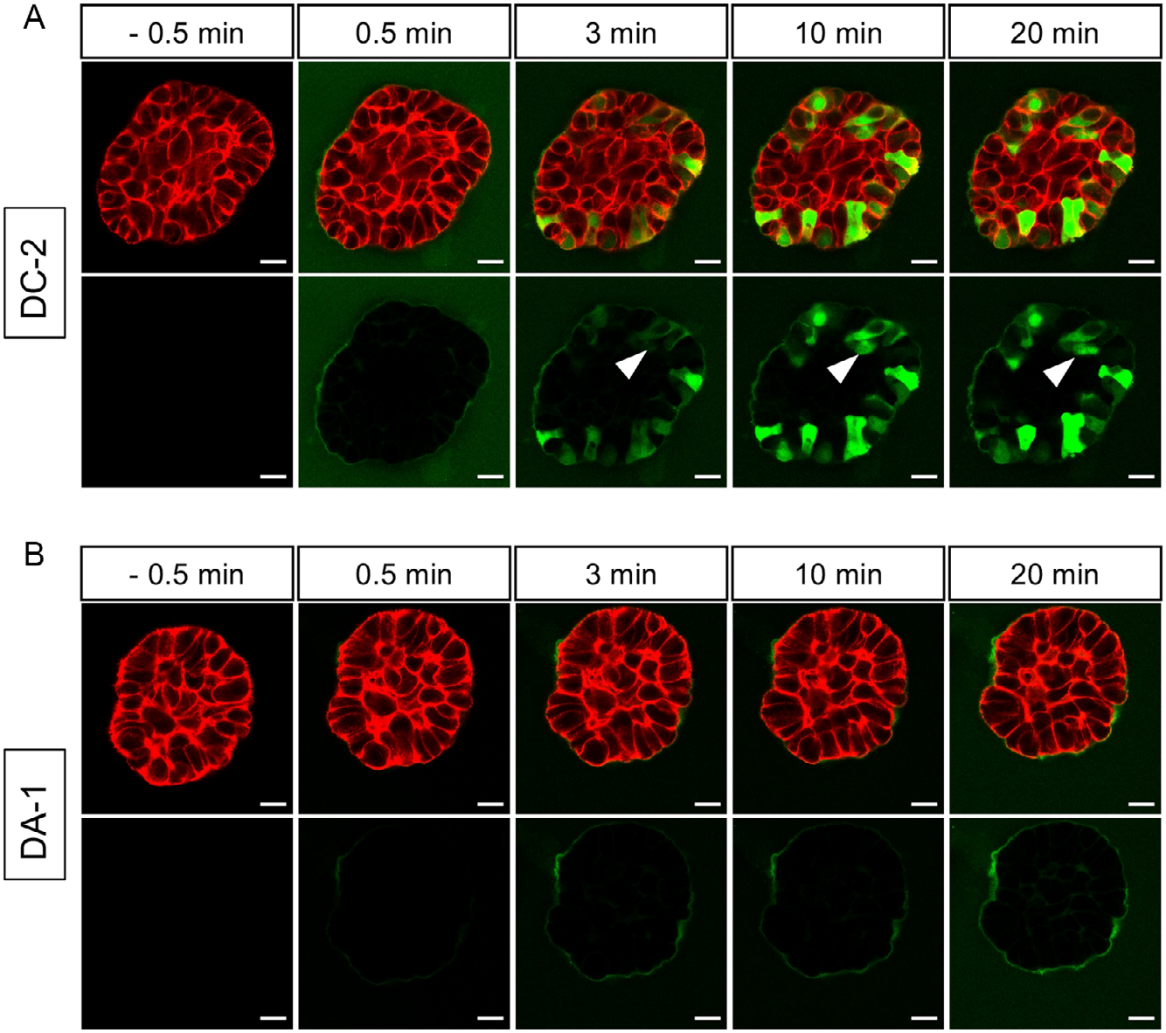
Confocal time‐lapse imaging of MCF‐10 A breast spheroid imaged at the equatorial plane. 10‐day‐old spheroids were treated with either A) 10 μM CF‐DC‐2 or B) 10 μM CF‐DA. Green: CF‐labeled peptide, red: F‐actin, scale bar: 20 μm. The experiment was performed *n* ≥ 2 with one n including 6 spheroids.

Afterwards, we investigated a more complex 3D cell system, i.e., human breast cell spheroids derived from non‐transformed MCF‐10 A cells. These basoapically polarizing spheroids develop an endogenous basement membrane (BM). This BM scaffold is made of a dense layer of extracellular matrix, which surrounds the spheroids and serves as a diffusion area for macromolecules.^[^ ^51^ ^]^ The BM consists of proteins such as collagen IV, glycoproteins including laminin and nidogen, as well as the highly negatively charged heparan sulfate proteoglycan perlecan, which strongly contributes to the overall negative net charge of the BM.^[^ ^52^, ^53^, ^54^ ^]^ Therefore, one would expect an entrapment in the BM of the cationic CPPs based on attraction by electrostatic interactions. This effect was indeed observed for the control peptide DA‐1, which quickly accumulated in the BM after an incubation time of 0.5–3 min (**Figure** **7**B and S10, Supporting Information) but did not further enter cells of the spheroid until the observed time point. However, after a first accumulation, DC‐2 efficiently surpassed the BM and translocated into many cells on different planes of the spheroids (Figure 7A and S10, Supporting Information), highlighting that the peptides successfully traversed the extracellular matrix. Moreover, we monitored that DC‐2 migrated from one cell into the next neighboring one (white arrows), proving that the peptides were indeed able to reach deeper layers of the spheroid. This was further verified by inspecting different focal image planes of the peptide‐treated spheroids (Figure S10, Supporting Information).

### 2.5 DC‐2 Alters Biological Function and Localization of Oncogenic Target Proteins

**Figure 8:**
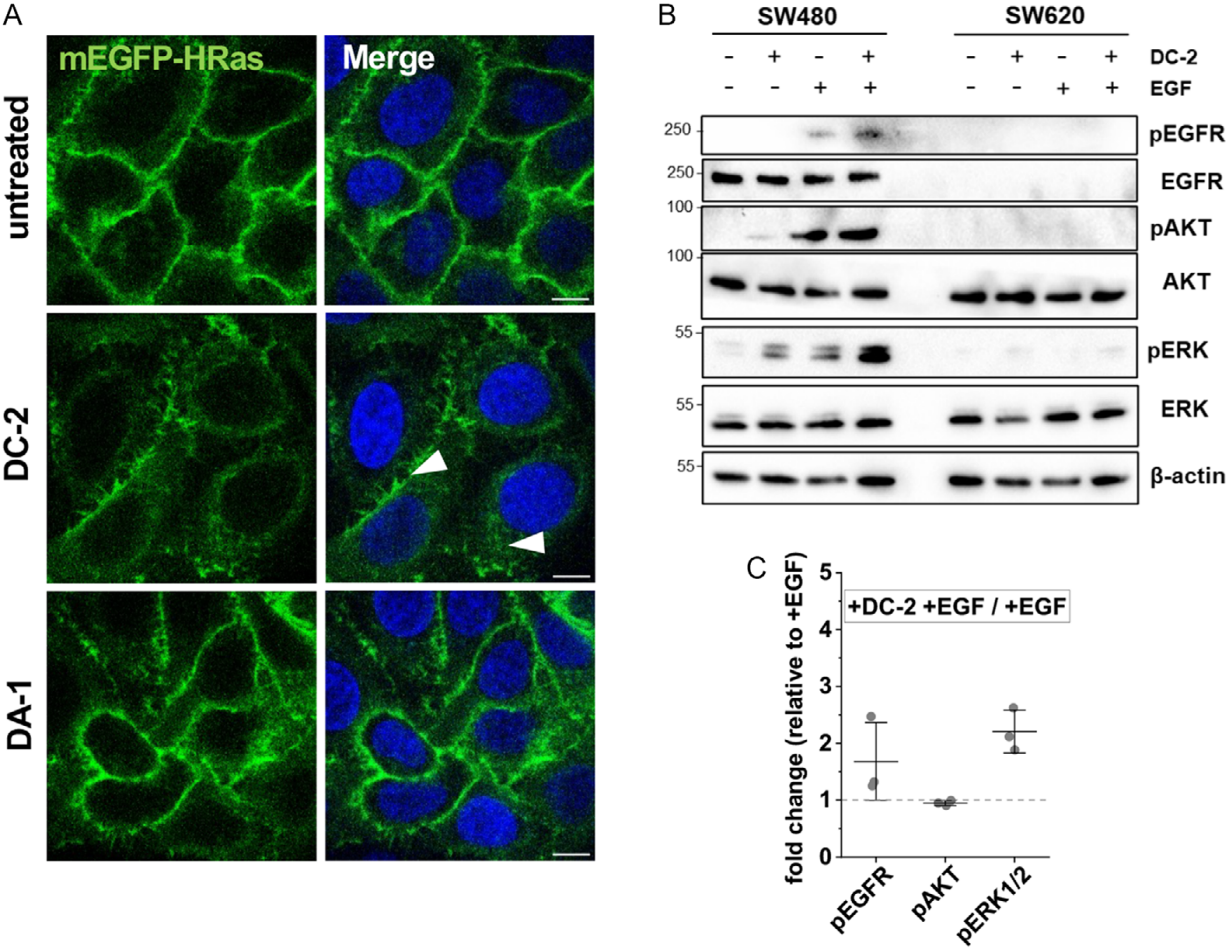
A) HeLa mEGFP‐HRas cells were induced overnight with doxycycline and treated with 100 μM DC‐2 or DA‐1 for 6 h (*n* = 3). Blue: Hoechst 33342 nuclear stain, scale bar: 10 μm. B) SW480 and SW620 cells were serum‐starved overnight and treated with 15 μM DC‐2 for 6 h followed by treatment with 200 ng ml^−1^ EGF for 15 min (*n* = 3). C) Fold change quantification of immunoblots normalized to β‐actin.

Finally, as a first proof of concept, we investigated if DC‐2 would alter the biological function, e.g., downstream signaling and subcellular localization of distinct target proteins having palmitoylation sites. For instance, we selected the small GTPase HRas, which is a dually palmitoylated protein. Usually, palmitoylation occurs at the Golgi apparatus by ZDHHC9, leading to the transport of HRas to the plasma membrane.^[^ ^22^, ^55^ ^]^ It was reported that in palmitoylation‐deficient HRas mutants, as well as when blocking protein palmitoylation, mislocalization of HRas to endomembranes is induced.^[^ ^56^, ^57^ ^]^ To analyze whether DC‐2 might also trigger this effect, we developed a transgenic HeLa cell line expressing monomeric enhanced green fluorescent protein (mEGFP)‐HRas. After 6 h peptide treatment with 100 μM DC‐2, we indeed observed an altered HRas pattern compared to untreated cells and to the control peptide DA‐1 (**Figure** **8**A). While some HRas proteins were still present at the membrane, an also uniform distribution throughout the cell (arrows) was detectable. To exclude any toxic effects of the peptides at these high concentrations, we also performed a cytotoxicity assay demonstrating that DC‐2 did not harm the cells when applied at 100 μM (Figure S11A, Supporting Information).

As a following test system, the receptor tyrosine kinase EGFR was chosen, which plays a crucial role in cancer. Amplification of EGFR and hyperactive EGFR mutants is associated with brain, lung, and breast cancer.^[^ ^58^, ^59^ ^]^ The C‐terminal tail of this receptor is palmitoylated, which is proposed to support association to the plasma membrane, leading to receptor inactivation and reduced MAPK signaling.^[^ ^21^, ^60^ ^]^ In fact, inhibiting palmitoylation by mutating the respective cysteines or silencing the respective palmitoyltransferase led to increased EGFR signaling.^[^ ^21^ ^]^ Therefore, we used this pathway to follow the influence of the DC‐2 peptide on EGFR signaling. As cell systems, we utilized human colorectal cancer cell lines SW480 and SW620, as they were previously reported to exhibit high (SW480) and low (SW620) EGFR expression (which we also verified in our assay) (Figure 8B).^[^ ^61^, ^62^ ^]^ After analyzing the biocompatibility of DC‐2 in the chosen cell lines, we decided to work with 15 μM peptide concentrations (Figure S11B,C, Supporting Information). Notably, when treating SW480 cells for 6 h with DC‐2 only, we observed a slight increase in ERK and AKT phosphorylation compared to untreated cells (Figure 8B). This finding was similar to the results reported by Runkle et al. using 2 BP in MDA‐MB‐231 cells.^[^ ^21^ ^]^ Moreover, an increase in EGFR and ERK phosphorylation was observed when treating SW480 cells with both DC‐2 and EGF compared to EGF only. Again, this agreed to what was recently reported by Runkle et al. (Figure 8B,C). As expected, no effects in SW620 cells were observed. Recently, it was described that a palmitoylation‐deficient variant of ERK2(C254A) shows significantly higher phosphorylation levels in presence of EGF compared to wild type ERK2, demonstrating a role of S‐palmitoylation also in the regulation of ERK phosphorylation.^[^ ^63^ ^]^ From this, we conclude a possible influence of DC‐2 on EGFR and its downstream signaling cascades, which may result from the peptides interfering with the palmitoylation of the individual signaling proteins. This observed impact on EGFR might be highly interesting to find novel strategies to treat cancers that are resistant to EGFR therapy and that might be then more susceptible to tyrosine kinase inhibitors.^[^ ^21^ ^]^

## 3 Conclusion

Taken together, we propose DC peptides as potential new tools to influence the S‐palmitoylation machinery, possibly by decreasing the available palmitoyl‐CoA pool. DC‐2 might be a potential candidate to affect the cellular localization and signaling pathways of oncogenic target proteins. EGFR has a relevant role in regulating and maintaining the biological characteristics of breast cancer cells, particularly in triple‐negative breast cancer.^[^ ^64^ ^]^ Here, we observed not only altering of EGFR signaling pathways induced by DC‐2 but also a highly efficient infiltration of MCF‐10 A‐derived human breast cell spheroids. Therefore, DC‐2 might be worth to be developed further in this cancer‐related context including cancers that are resistant to EGFR therapy. Probably, detailed future studies will additionally reveal peptide tools able to affect not only the herein presented but probably also other palmitoylated oncogenes.

## 4 Experimental Section

4.1

4.1.1

##### Peptide Synthesis and Purification

Peptides were synthesized on a Rinkamide resin using an automated solid phase peptide synthesis (SPPS) synthesizer Syro I (MultiSyntech) according to the Fmoc/tBu strategy as previously described.^[^ ^65^ ^]^ For fluorescent detection, all peptides were N‐terminally labeled with CF by incubating the fully protected peptide overnight at room temperature (RT) with 5 equivalents of each CF, oxyma, and *N,N*′‐diisopropylcarbodiimide dissolved in DMF. To increase the coupling efficiency, the fully protected peptide was incubated for 2 h at RT with 5 equivalents of each CF, (O‐(7‐Azabenzotriazol‐1‐yl)‐N,N,N′,N′‐tetramethyluronium‐hexafluorphosphate), and *N,N′*‐diisopropylethylamine dissolved in DMF. A mixture of trifluoroacetic acid (TFA)/thioanisole/1,2‐ethanedithiol (90:7:3, v/v/v) was added for 3 h under shaking conditions to cleave the peptide from the resin and remove all protection groups. The peptides were precipitated in diethyl ether and lyophilized. For the first biological screen, crude peptides were used except for CF‐DC‐4, CF‐DC‐6, CF‐DC‐8, CF‐DC‐10, CF‐DC‐11, CF‐DC‐13, and CF‐sC18* that were purified using a preparative reversed phase‐high pressure liquid chromatography (RP‐HPLC) (Hitachi Elite LaChrom) on a VP250/16 NUCLEODUR 100‐5 C18ec column (Macherey Nagel) using linear gradients from 20% to 70% B in A (A = 0.1% TFA in water; B = 0.1% TFA in acetonitrile [ACN]) over 60 min. All analytical data were measured on an electrospray‐ionization mass spectrometer (ESI‐MS) LTQ‐XL (Thermo Scientific) using a gradient of 10 to 60% of ACN in ddH_2_O + 0.1% formic acid (FA) on an Aeris 3.6 μm PEPTIDE XB‐C18 100 LC column (Phenomenex). A 1 mM stock solution of the final peptide was dissolved in ddH_2_O.

##### Secondary Structure Analysis

CD spectroscopy was performed on the spectropolarimeter J 715 (Jasco) using either 20 μM peptide in 10 mM phosphate buffer (pH 7.0) or 20 μM peptide in 10 mM phosphate buffer (pH 7.0)/trifluoroethanol (1:1, v/v). The ellipticity [mdeg] of each peptide was recorded in triplicates from 180 to 260 nm. The following instrumental settings were used during the measurement: 100 mdeg sensitivity, 0.2–1 nm data pitch, 100 nm min^−1^ scanning speed, 2 s response, and a bandwidth of 1.0 nm. Both buffer solutions were recorded as blank measurements and subtracted from the peptide measurements and all measurements were smoothed. The ellipticity was then converted into the molar ellipticity [Θ] using the following formula:

(1) $$[\Theta ]=\frac{{\left[\Theta \right]}_{\text{measured}}}{c^{*}n^{*}l^{*}10}$$

where c indicates concentration, n indicates number of amino acids, and l indicates length of cuvette.

##### Synthesis of Peptide Disulfide

For generating the disulfide, the crude lyophilized peptide was used. And, 50 μM of peptide was dissolved in 10 mM ammonium bicarbonate buffer (pH 8.0); 0.025% H_2_O_2_ was added, and the mixture was shaken for up to 2.5 min; and 500 μl TFA was added to stop the reaction, and a solid‐phase extraction using an solid phase extraction cartridge (Chromafix) was performed to get rid of the salts in the buffer. Finally, the peptide was purified by preparative HPLC. The final peptides were analyzed on an analytical HPLC ESI‐MS (LTQ XL, Thermo Scientific) and a 1 mM stock solutions was prepared in ddH_2_O.

##### Cell Culture

Cell lines were cultured in RPMI medium (HeLa) or DMEM medium (MDA‐MB‐231, HFF‐1, HeLa Flip‐In, SW480, SW620) supplemented with 10% fetal bovine serum and 4 mM L‐glutamine at 37 °C humidified atmosphere with 5% CO_2_. Upon reaching 80–90% confluency, cells were split using 0.5 mg ml^−1^ trypsin/EDTA or seeded for cellular assays. MCF‐10 A cells for spheroid morphogenesis were transduced with red fluorescent protein (RFP)‐LifeAct (IBIDI). The cells were maintained under standard culture conditions (37 °C, 5% CO_2_) in DMEM/F12 growth medium containing 5% horse serum, 0.5 μg mL^−1^ hydrocortisone, 100 ng mL^−1^ cholera toxin, 20 ng mL^−1^ EGF, 10 μg mL^−1^ insulin, 100 U mL^−1^ penicillin, and 100 μg mL^−1^ streptomycin.

##### Cell Viability Assay

HeLa (13,000), MDA‐MB‐231 (26,000), HFF‐1 (16,000) cells, mEGFP‐HRas (18,000), and SW480 and SW620 (15,000) were seeded into a 96‐well plate. Cells were treated with suitable peptide concentrations diluted in the corresponding serum‐free culture medium. After 24 h incubation under standard growth conditions, the cells were washed once with PBS. The positive control was treated for 10 min with 70% EtOH. To test the viability, cells were treated with a resazurin solution (In Vitro Toxicology Assay Kit, resazurin based, Sigma) and incubated for a further 1–2 h. The product resorufin was measured at 595 nm (λex = 550 nm) on an infinite M200 plate reader (Tecan) and normalized to the untreated negative control cells.

##### Flow Cytometry

HeLa (90,000), MDA‐MB‐231 (120,000), and HFF‐1 (125,000) cells were seeded into a 24‐well plate. Cells were treated with the desired concentration of CF‐labeled peptide diluted in the appropriate serum‐free medium for 30 min at 37 °C and 5% CO_2_. Afterward, cells were washed once with PBS, detached from the plate using indicator‐free trypsin/EDTA and resuspended in indicator‐free culture medium. The cell mixture was measured on the Guava easyCyte flow cytometer (Merck) using the GRN‐B (520/30) detector.

CMA was synthesized and kindly provided by Azizi et al.^[^ ^33^ ^]^ Cells were either treated with 20 μM CMA diluted in serum‐free media or 0.1% DMSO diluted in serum‐free media for 6 h at 37 °C with 5% CO_2_. Next, cells were washed once with serum‐free medium and 5 μM CF‐labeled peptides diluted in serum‐free medium were incubated for 30 min. Control cells were only treated with dimethylsulfoxide (DMSO) or 20 μM CMA.

For the preincubation assay, cells were either treated with 5 μM DC‐2 diluted in serum‐free medium or only serum‐free medium for 30 min at 37 °C and 5% CO_2_. Afterward, cells were washed once with serum‐free medium and treated with 5 μM CF‐labeled DC‐2 for 30 min at 37 °C and 5% CO_2_.

To block cell surface thiols, cells were incubated with either serum‐free medium or 1.2 mM 5,5′‐dithiobis‐(2‐nitrobenzoic acid) (Ellman's reagent, DTNB) diluted in serum‐free medium for 5 min. Next, untreated cells were treated with 1 μM of CF‐labeled DC‐2 or DC‐2_dis_ diluted in serum‐free medium and DTNB‐pretreated cells with 1 μM of CF‐labeled DC‐2 or DC‐2_dis_ and 1.2 mM DTNB in serum‐free medium for 30 min at 37 °C and 5% CO_2_ as described by Aubry et al.^[^ ^47^ ^]^

##### Live‐Cell Fluorescence Imaging

HeLa (40,000), MDA‐MB‐231 (30,000), or HFF1 (55,000) cells were seeded into an 8‐well ibidi. Cells were treated for 30 min with 5 μM of CF‐labeled peptide diluted in the appropriate serum‐free media. The cell nuclei were stained with the Hoechst 33342 nuclear dye for the last 10 min of the total incubation time. Afterward, cells were washed three to four times using serum‐free medium and finally, medium containing FBS was added for the microscopy. Live fluorescence microscopy analyses were performed on the LSM 980 with Airyscan 2 Inverse Confocal Laser Scanning Microscope (Carl Zeiss) on a 63X Plan‐Apochromat oil objective (NA 1.4, Zeiss) at 37 °C and 5% CO_2_ or on the BZ‐X810 (Keyence) using a 60X Plan‐Apochromat oil objective (NA 1.4, Keyence). Images were cropped and processed using Fiji.

##### MCF‐10 A Spheroid Morphogenesis and Isolation from EHS Matrix

Spheroids from single MCF10‐A cells were generated for 10 days as described in ref. [66]. For further analyses, spheroids were isolated from EHS matrix through incubation in 2 mL ice‐cold cell recovery solution (BD Biosciences) for 30 min (4 °C). Next, spheroids were washed with fresh EGF‐free medium and individually picked under a stereo microscope for seeding onto 35 mm cell culture dishes with microscopic glass bottoms (Cover Slip, 24 × 24 mm, #1.5 HP, Menzel‐Gläser). Spheroids adhered to glass substrates for 15 min (RT) and were subsequently covered with 0.9 mL fresh EGF‐free growth medium.

##### Confocal Microscopy of MCF‐10 A Spheroids

Live‐cell imaging was performed at 37 °C and 5% CO_2_ (cell incubator XL, Zeiss, Germany) with an inverse confocal laser scanning microscope (LSM880 with Airyscan detector) that used a 40x LD C‐Apochromat water immersion objective (NA 1.1, Zeiss). Before addition of peptides, control images were taken. Subsequently, peptide was added to a final concentration of 10 μM and spheroids immediately imaged every 45 s for 20 min. The observed plane was at the equatorial plane of a spheroid, where the diameter was largest. Images were taken with Fast Airyscan and subsequent processing using the ZEN 2.3 black software (Zeiss). Z‐stacks were taken right after the time series in 1 μm steps with the same microscopic settings.

##### Co‐localization Studies

HeLa cells (30,000–40,000) were seeded into an 8‐well ibidi. For lysosomal and lipid droplet analysis, cells were treated for 30 min with 5 μM of CF‐labeled peptide combined with either 100 nM LysoTracker Red DND‐99 (Invitrogen) or a 1:1000 solution of distyryl Bodipy lipid droplet stain synthesized and kindly provided by Engelhardt et al.^[^ ^67^ ^]^ diluted in serum‐free media under standard growth conditions. For Golgi analysis, cells were treated for 2 h with 5 μM CF‐labeled peptide under standard growth conditions. For the last 30 min of the incubation period, 1:100 BODIPY TR ceramide complexed to bovine serum albumine (BSA) (Invitrogen) was directly diluted into the well and incubated for the last 30 min together with the peptide. For ER staining, CellLight ER‐RFP, BacMam 2.0 (Invitrogen), was added to the cells and incubated overnight under standard growth conditions. The next day, the cells were treated for 2 h with 5 μM CF‐labeled peptides as described earlier. After the peptide and stain incubation, cells were washed three to four times with serum‐free medium. Finally, complete medium was added, and cells were imaged on the Ultra‐View VoX Spinning Disk Confocal Microscope (Perkin Elmer) on a Plan‐Apo Tirf 60X oil objective (NA 1.49, Nikon) at 37 °C and 5% CO_2_. Images were cropped and processed using Fiji. Pearson and Manders’ Coefficients were calculated using the JaCoP analysis tool.^[^ ^68^ ^]^ For Golgi analysis, no PCC or MOC was determined as the unedited images contained a strong background signal.

##### In Vitro Palmitoylation Assay

The 50 μM CF‐labeled peptides were incubated with 100 nM human 6His‐ZDHHC7 (Proteintech) and 100 μM palmitoyl‐CoA (Santa Cruz Biotechnology) in reaction buffer (50 mM HEPES, 150 mM NaCl, 1 mM EDTA, 1 mM (tris(2‐carboxyethyl)phosphine) (TCEP), 0.3 mM DDM, pH 7.0) for 2 h at 37 °C. Control reactions without ZDHHC7 or palm‐CoA were incubated with the appropriate volume of reaction buffer. Afterward, the reaction was centrifuged for 5 min at 13000 rpm. The precipitate was dissolved in DMSO/ACN (3:2, v/v). To enrich the peptide from the supernatant, C8 ZipTips were used. All samples were analyzed on the LC MS‐8060 triple quadrupole ESI‐MS (Shimadzu). The fluorescence intensity (λex = 493 nm, λem = 517 nm) of the samples was measured on an EC 160/2 NUCLEODUR 300‐5 C18 ec column using linear gradients from 20% to 70 % ACN in ddH_2_O + 0.1% FA over 7 min.

##### Generating mEGFP‐HRas Cell Line

mEGFP‐HRas was amplified from a plasmid gifted from Karel Svoboda^[^ ^69^ ^]^ (Addgene plasmid # 18662) using the primers 5′‐gcgGGTACCgccaccatggtgagcaagg‐3′ and 5′‐gcgGCGGCCGCtcaggagagcacacactt‐3′. KpnI and NotI restriction sites (capital letter) were used and the construct was cloned into the pcDNA5 FRT‐TO vector to generate an inducible T‐REx‐HeLa cell line using the Flp‐In T‐REx System (Invitrogen). The plasmid was verified by sequencing. The construct was co‐transfected together with the pOG44 vector using the FuGENE HD transfection reagent (Promega). Positive clones were selected with DMEM medium supplemented with 10% FCS, 500 μg ml^−1^ penicillin/streptomycin 100 μg ml^−1^ hygromycin and 10 μg ml^−1^ blasticidin and verified by fluorescence microscopy and immunoblot.

##### Fluorescence Microscopy Using mEGFP‐HRas Expressing Cells

A total of 35,000 HeLa mEGFP‐HRas cells were seeded into an 8‐well plate. Cells were induced with 1 mg ml^−1^ doxycycline and incubated for around 15 h under standard growth conditions. Afterward, cells were treated for 6 h with 100 μM peptide diluted in serum‐free medium. And, 10 min prior to the end of the incubation time, the Hoechst 33342 nuclear dye was added. Cells were washed with serum‐free media. Finally, complete medium was added and cells were imaged on the UltraView VoX Spinning Disk Confocal Microscope (Perkin Elmer) on a 60X oil objective at 37 °C and 5% CO_2_. Images were cropped and processed using Fiji.

##### EGFR‐Related Signaling Cascade

SW480 and SW620 cells (1,200,000) were seeded into a 6‐well plate. The next day, cells were once washed with PBS and starved for 18 h with DMEM + 0.2% BSA. Next, 15 μM DC‐2 diluted in DMEM + 0.2% BSA was incubated with the cells for 6 h. Control wells were only treated with the starvation medium. Afterward, all wells were washed once with PBS and 200 ng ml^−1^ EGF diluted in DMEM + 0.2% BSA was added for 15 min. Cells were again washed once with PBS and ice‐cold lysis buffer (25 mM Tris, 150 mM NaCl,1 mM TCEP, 2 mM EDTA, 1% Triton‐X‐100, pH 7.4) supplemented with Halt Protease and Phosphatase Inhibitor Cocktail (Thermo Fisher) was added to scrape the cells from the well. Lysates were incubated on ice for 30 min slightly shaking. After centrifugation for 30 min at 14,000 rpm at 4 °C, lysates were mixed with 4X SDS‐PAGE loading buffer (200 mM Tris‐HCl pH 6.8, 400 mM dithiothreitol, 8% SDS, 0.4% bromophenol blue, and 40% glycerol) and heated at 95 °C for 5 min. Protein samples were analyzed by SDS‐PAGE and immunoblotting. The following antibodies were used: anti‐EGFR (Cell Signaling Technology (CST) Cat#4267), anti‐pEGFR (CST Cat #3777), anti‐AKT (CST Cat#9272); anti‐pAKT (CST Cat#4060); anti‐ERK1/2 (CST Cat#4695); anti‐pERK1/2 (CST Cat#4370); anti‐β‐actin‐horseradish peroxidase (HRP) (Santa Cruz Biotechnology Cat# sc‐47778 HRP); or anti‐rabbit‐HRP conjugate (CST Cat#7074S). Images were cropped, processed, and quantified using the ImageLab software (BioRad).

## Conflict of Interest

The authors declare no conflict of interest.

## Supporting information

Supplementary Material

## Data Availability

The data that support the findings of this study are available from the corresponding author upon reasonable request.

## References

[1] M. Blanc , F. P. A. David , F. G. van der Goot , Methods Mol. Biol., Humana Press Inc. 2019, pp. 203–214. 10.1007/978-1-4939-9532-5_16 31152406

[2] F. S. Mesquita , L. Abrami , M. E. Linder , S. X. Bamji , B. C. Dickinson , F. G. van der Goot , Nat. Rev. Mol. Cell Biol. 2024, 25, 488. 38355760 10.1038/s41580-024-00700-8 PMC12010433

[3] S. Tabaczar , A. Czogalla , J. Podkalicka , A. Biernatowska , A. F. Sikorski , Exp. Biol. Med. 2017, 242, 1150. 10.1177/1535370217707732 PMC5478004 28485685

[4] Y. Ohno , A. Kihara , T. Sano , Y. Igarashi , Biochim. Biophys. Acta, Mol. Cell Biol. Lipids 2006, 1761, 474. 10.1016/j.bbalip.2006.03.010 16647879

[5] M. S. Rana , C. J. Lee , A. Banerjee , Biochem. Soc. Trans. 2019, 47, 157. 30559274 10.1042/BST20180429

[6] B. C. Jennings , M. E. Linder , J. Biol. Chem. 2012, 287, 7236. 22247542 10.1074/jbc.M111.337246 PMC3293542

[7] H. Hou , A. T. John Peter , C. Meiringer , K. Subramanian , C. Ungermann , Traffic 2009, 10, 1061. 19453970 10.1111/j.1600-0854.2009.00925.x

[8] D. A. Mitchell , G. Mitchell , Y. Ling , C. Budde , R. J. Deschenes , J. Biol. Chem. 2010, 285, 38104. 20851885 10.1074/jbc.M110.169102 PMC2992244

[9] L. A. Camp , S. L. Hofmann , J. Biol. Chem. 1993, 268, 22566. 7901201

[10] J. A. Duncan , A. G. Gilman , J. Biol. Chem. 1998, 273, 15830. 9624183 10.1074/jbc.273.25.15830

[11] A. A. Soyombo , S. L. Hofmann , J. Biol. Chem. 1997, 272, 27456. 9341199 10.1074/jbc.272.43.27456

[12] N. Yokoi , Y. Fukata , A. Sekiya , T. Murakami , K. Kobayashi , M. Fukata , J. Neurosci. 2016, 36, 6431. 27307232 10.1523/JNEUROSCI.0419-16.2016 PMC5015780

[13] Y. Cao , T. Qiu , R. S. Kathayat , S. A. Azizi , A. K. Thorne , D. Ahn , Y. Fukata , M. Fukata , P. A. Rice , B. C. Dickinson , Nat. Chem. Biol. 2019, 15, 1232. 31740833 10.1038/s41589-019-0399-y PMC6871660

[14] D. T. S. Lin , E. Conibear , Elife 2015, 4, e11306. 26701913 10.7554/eLife.11306 PMC4755737

[15] V. M. Tomatis , A. Trenchi , G. A. Gomez , J. L. Daniotti , PLoS One 2010, 5, 15045. 10.1371/journal.pone.0015045 PMC2994833 21152083

[16] O. Rocks , M. Gerauer , N. Vartak , S. Koch , Z. P. Huang , M. Pechlivanis , J. Kuhlmann , L. Brunsveld , A. Chandra , B. Ellinger , H. Waldmann , P. I. H. Bastiaens , Cell 2010, 141, 458. 20416930 10.1016/j.cell.2010.04.007

[17] P. Ko , S. J. Dixon , EMBO Rep. 2018, 19, e46666. 30232163 10.15252/embr.201846666 PMC6172454

[18] J. Jin , X. Zhi , X. Wang , D. Meng , J. Cell Physiol. 2021, 236, 3220. 33094504 10.1002/jcp.30122

[19] B. Chen , B. Zheng , M. Deran , G. K. Jarugumilli , J. Fu , Y. S. Brooks , X. Wu , Nat. Chem. Biol. 2016, 12, 686. 27380321 10.1038/nchembio.2119 PMC4990496

[20] D. T. Coleman , A. L. Gray , S. J. Kridel , J. A. Cardelli , Oncotarget 2016, 7, 32664. 27081699 10.18632/oncotarget.8706 PMC5078042

[21] K. B. Runkle , A. Kharbanda , E. Stypulkowski , X. J. Cao , W. Wang , B. A. Garcia , E. S. Witze , Mol. Cell 2016, 62, 385. 27153536 10.1016/j.molcel.2016.04.003 PMC4860254

[22] C. Busquets‐Hernández , G. Triola , Front. Mol. Biosci. 2021, 8, 659861. 33816563 10.3389/fmolb.2021.659861 PMC8010249

[23] B. Cuiffo , R. Ren , Blood 2010, 115, 3598. 20200357 10.1182/blood-2009-03-213876 PMC2867268

[24] J. Tang , W. Peng , Y. Feng , X. Le , K. Wang , Q. Xiang , L. Li , Y. Wang , C. Xu , J. Mu , K. Xu , P. Ji , Q. Tao , A. Huang , C. X. Deng , Y. Lin , T. Xiang , Oncogene 2021, 40, 5416. 34282274 10.1038/s41388-021-01949-5 PMC8413129

[25] M. Yeste‐Velasco , X. Mao , R. Grose , S. C. Kudahetti , D. Lin , J. Marzec , N. Vasiljević , T. Chaplin , L. Xue , M. Xu , J. M. Foster , S. S. Karnam , S. Y. James , A. M. Chioni , D. Gould , A. T. Lorincz , R. T. D. Oliver , C. Chelala , G. M. Thomas , J. M. Shipley , S. J. Mather , D. M. Berney , B. D. Young , Y. J. Lu , J. Pathol. 2014, 232, 566. 24407904 10.1002/path.4327

[26] C. E. Ducker , E. M. Stettler , K. J. French , J. J. Upson , C. D. Smith , Oncogene 2004, 23, 9230. 15489887 10.1038/sj.onc.1208171 PMC2908390

[27] C. E. Ducker , L. K. Griffel , R. A. Smith , S. N. Keller , Y. Zhuang , Z. Xia , J. D. Diller , C. D. Smith , Mol. Cancer Ther. 2006, 5, 1647. 16891450 10.1158/1535-7163.MCT-06-0114 PMC2888271

[28] M. S. Rana , P. Kumar , C. J. Lee , R. Verardi , K. R. Rajashankar , A. Banerjee , Science 2018, 359, eaao6326. 29326245 10.1126/science.aao6326 PMC6317078

[29] T. Lan , C. Delalande , B. C. Dickinson , Curr. Opin. Chem. Biol. 2021, 65, 118. 34467875 10.1016/j.cbpa.2021.07.002 PMC8671176

[30] M. P. Pedro , A. A. Vilcaes , V. M. Tomatis , R. G. Oliveira , G. A. Gomez , J. L. Daniotti , PLoS One 2013, 8, e75232. 24098372 10.1371/journal.pone.0075232 PMC3788759

[31] R. A. Coleman , P. Rao , R. J. Fogelsong , E. S. G. Bardes , Biochim. Biophys. Acta 1992, 1125, 203. 1571364 10.1016/0005-2760(92)90046-x

[32] D. Davda , M. A. El Azzouny , C. T. M. B. Tom , J. L. Hernandez , J. D. Majmudar , R. T. Kennedy , B. R. Martin , ACS Chem. Biol. 2013, 8, 1912. 23844586 10.1021/cb400380s PMC3892994

[33] S.‐A. Azizi , T. Lan , C. Delalande , R. S. Kathayat , F. Banales Mejia , A. Qin , N. Brookes , P. J. Sandoval , B. C. Dickinson , ACS Chem. Biol. 2021, 16, 1546. 34309372 10.1021/acschembio.1c00405 PMC8590885

[34] A. Klimpel , K. Stillger , J. L. Wiederstein , M. Krüger , I. Neundorf , FEBS J. 2021, 288, 2911. 33112492 10.1111/febs.15612

[35] M. Horn , F. Reichart , S. Natividad‐Tietz , D. Diaz , I. Neundorf , Chem. Commun. 2016, 52, 2261. 10.1039/c5cc08938g 26725983

[36] A. Gronewold , M. Horn , I. Neundorf , Beilstein J. Org. Chem. 2018, 14, 1378. 29977402 10.3762/bjoc.14.116 PMC6009097

[37] H. C. Hymel , A. Rahnama , O. M. Sanchez , D. Liu , T. J. Gauthier , A. T. Melvin , Cells 2022, 11, 1195. 35406759 10.3390/cells11071195 PMC8997848

[38] A. Vahedi , P. Bigdelou , A. M. Farnoud , Sci. Rep. 2020, 10, 1. 32934292 10.1038/s41598-020-72176-3 PMC7492248

[39] E. D. Timotievich , I. P. Shilovskiy , M. R. Khaitov , Biochemistry 2023, 88, 1800. 38105200 10.1134/S0006297923110111

[40] J. Grabeck , T. Lützenburg , P. Frommelt , I. Neundorf , Molecules 2022, 27, 6656. 36235193 10.3390/molecules27196656 PMC9570898

[41] M. Hao , L. Zhang , P. Chen , Int. J. Mol. Sci. 2022, 23, 9038. 36012300 10.3390/ijms23169038 PMC9409441

[42] J. Hoon Oh , S.‐E. Chong , S. Nam , S. Hyun , S. Choi , H. Gye , S. Jang , J. Jang , S. Won Hwang , J. Yu , Y. Lee , J. H. Oh , S. Chong , S. Nam , S. Choi , S. Jang , J. Jang , Y. Lee , S. Hyun , J. Yu , H. Gye , S. W. Hwang , Adv. Sci. 2018, 5, 1800240. 10.1002/advs.201800240 PMC6096998 30128238

[43] M. Klußmann , K. Stillger , M. Ruppel , C. Sticker , I. Neundorf , J. Pept. Sci. 2024, 30, e3604. 38651525 10.1002/psc.3604

[44] M. Hällbrink , A. Florén , A. Elmquist , M. Pooga , T. Bartfai , Ü. Langel , Biochim. Biophys. Acta, Biomembr. 2001, 1515, 101. 10.1016/s0005-2736(01)00398-4 11718666

[45] A. F. L. Schneider , A. L. D. Wallabregue , L. Franz , C. P. R. Hackenberger , Bioconjug. Chem. 2019, 30, 400. 30616339 10.1021/acs.bioconjchem.8b00855

[46] T. Li , W. Gao , J. Liang , M. Zha , Y. Chen , Y. Zhao , C. Wu , Anal. Chem. 2017, 89, 8501. 28714307 10.1021/acs.analchem.7b02084

[47] S. Aubry , F. Burlina , E. Dupont , D. Delaroche , A. Joliot , S. Lavielle , G. Chassaing , S. Sagan , FASEB J. 2009, 23, 2956. 19403512 10.1096/fj.08-127563

[48] Q. Laurent , R. Martinent , B. Lim , A. T. Pham , T. Kato , J. López‐Andarias , N. Sakai , S. Matile , JACS Au 2021, 1, 710. 34467328 10.1021/jacsau.1c00128 PMC8395643

[49] I. Neundorf , R. Rennert , J. Hoyer , F. Schramm , K. Löbner , I. Kitanovic , S. Wölfl , Pharmaceuticals 2009, 2, 49. 27713223 10.3390/ph2020049 PMC3978507

[50] F. Duchardt , M. Fotin‐Mleczek , H. Schwarz , R. Fischer , R. Brock , Traffic 2007, 8, 848. 17587406 10.1111/j.1600-0854.2007.00572.x

[51] A. Gaiko‐Shcherbak , G. Fabris , G. Dreissen , R. Merkel , B. Hoffmann , E. Noetzel , PLoS One 2015, 10, e0145174. 26674091 10.1371/journal.pone.0145174 PMC4684506

[52] R. Kalluri , Nat. Rev. Cancer 2003, 3, 422. 12778132 10.1038/nrc1094

[53] K. M. Mak , R. Mei , Anat. Rec. 2017, 300, 1371. 10.1002/ar.23567 28187500

[54] J. H. Miner , Exp. Cell. Res. 2012, 318, 973. 22410250 10.1016/j.yexcr.2012.02.031 PMC3334451

[55] J. T. Swarthout , S. Lobo , L. Farh , M. R. Croke , W. K. Greentree , R. J. Deschenes , M. E. Linder , J. Biol. Chem. 2005, 280, 31141. 16000296 10.1074/jbc.M504113200

[56] S. Roy , S. Plowman , B. Rotblat , I. A. Prior , C. Muncke , S. Grainger , R. G. Parton , Y. I. Henis , Y. Kloog , J. F. Hancock , Mol. Cell Biol. 2005, 25, 6722. 16024806 10.1128/MCB.25.15.6722-6733.2005 PMC1190337

[57] J. S. Goodwin , K. R. Drake , C. Rogers , L. Wright , J. Lippincott‐Schwartz , M. R. Philips , A. K. Kenworthy , J. Cell Biol. 2005, 170, 261. 16027222 10.1083/jcb.200502063 PMC2171405

[58] M. L. Uribe , I. Marrocco , Y. Yarden , Cancers 2021, 13, 2748. 34206026 10.3390/cancers13112748 PMC8197917

[59] H. Masuda , D. Zhang , C. Bartholomeusz , H. Doihara , G. N. Hortobagyi , N. T. Ueno , Breast Cancer Res. Treat. 2012, 136, 331. 23073759 10.1007/s10549-012-2289-9 PMC3832208

[60] Y. A. Kadry , J.‐Y. Lee , E. S. Witze , Open Biol. 2021, 11, 210033. 34610265 10.1098/rsob.210033 PMC8492172

[61] R. Sun , Y. Zhu , H. Feng , Z. Yang , X. Bian , P. Gu , C. Wang , Y. Liu , Cell Physiol. Biochem. 2015, 35, 1877. 25871436 10.1159/000373998

[62] M. Goetz , A. Ziebart , S. Foersch , M. Vieth , M. J. Waldner , P. Delaney , P. R. Galle , M. F. Neurath , R. Kiesslich , Gastroenterology 2010, 138, 435. 19852961 10.1053/j.gastro.2009.10.032

[63] S. A. Azizi , T. Qiu , N. E. Brookes , B. C. Dickinson , Cell Rep. 2023, 42, 113135. 37715953 10.1016/j.celrep.2023.113135 PMC10591828

[64] X. Li , L. Zhao , C. Chen , J. Nie , B. Jiao , Biochim. Biophys. Acta, Rev. Cancer 2022, 1877, 188789. 36064121 10.1016/j.bbcan.2022.188789

[65] I. Raote , A. H. Rosendahl , H. M. Häkkinen , C. Vibe , I. Küçükaylak , M. Sawant , L. Keufgens , P. Frommelt , K. Halwas , K. Broadbent , M. Cunquero , G. Castro , M. Villemeur , J. Nüchel , A. Bornikoel , B. Dam , R. K. Zirmire , R. Kiran , C. Carolis , J. Andilla , P. Loza‐Alvarez , V. Ruprecht , C. Jamora , F. Campelo , M. Krüger , M. Hammerschmidt , B. Eckes , I. Neundorf , T. Krieg , V. Malhotra , Nat. Commun. 2024, 15, 1. 38658535 10.1038/s41467-024-47004-1 PMC11043333

[66] J. Eschenbruch , G. Dreissen , R. Springer , J. Konrad , R. Merkel , B. Hoffmann , E. Noetzel , Cells 2021, 10, 1979. 34440749 10.3390/cells10081979 PMC8394122

[67] P. M. Engelhardt , M. Veronese , A. A. Eryiğit , A. Das , A. T. Kaczmarek , E. I. Rugarli , H. G. Schmalz , Chem. ‐ Eur. J. 2024, 30, e202400808. 38506349 10.1002/chem.202400808

[68] S. Bolte , F. P. Cordelières , J. Microsc. 2006, 224, 213. 17210054 10.1111/j.1365-2818.2006.01706.x

[69] R. Yasuda , C. D. Harvey , H. Zhong , A. Sobczyk , L. Van Aelst , K. Svoboda , Nat. Neurosci. 2006, 9, 283. 16429133 10.1038/nn1635

