# Supplementary material for: First Steps toward the Design of Peptides that Influence the Intracellular Palmitoylation Machinery
Source: Chembiochem. 2025 Apr 23;26(10):e202500218. doi: 10.1002/cbic.202500218 (PMC12117998; doi:10.1002/cbic.202500218)
Supplement: Supplementary file 1 — Supplementary Material [file CBIC-26-e202500218-s001.zip › Cbic.202500218-sup-0001-suppdata-S1.pdf]

Supporting Information

First steps towards the design of peptides that influence the intracellular palmitoylation machinery

Katharina Stillger[a], Eric Platz-Baudin[b], Florian Friedland[b], Melina Ruppel[a], Coco-Louisa Sticker[a], Anne Bodenhausen[a], Eric Noetzel[b] and Ines Neundorf*[a]

[a] Dr. K. Stillger, M. Ruppel, C.-L. Sticker, A. Bodenhausen, Prof. Dr. I. Neundorf
Institute for Biochemistry, Department of Chemistry and Biochemistry
University of Cologne
Zülpicher Straße 47, 50674 Cologne, Germany
E-mail: ines.neundorf@uni-koeln.de

[b] E. Platz-Baudin, Dr. F. Friedland, Dr. E. Noetzel
Institute of Biological Information Processing 2: Mechanobiology
Forschungszentrum Jülich
Wilhelm-Johnen-Straße, 52428, Jülich, Germany

Table of contents

Supplementary Methods 2
Supplementary Figures 4

Supplementary Methods

Membrane integrity assay

Hela cells (15,000) were seeded into a 96-well plate. Cells were treated with suitable peptide concentrations diluted in the corresponding serum-free culture medium. After 1 h peptide incubation under standard growth conditions, the positive control was either directly treated with lysis solution or the supernatant was exchanged for serum-free culture medium and the plate was incubated for further 5 h under growing conditions. The CytoTox-ONE™ Reagent was added and incubated at RT for 10 minutes. Finally, the Stop Solution was added and the fluorescence was measured at 595 nm (λ_{ex} = 550 nm) on an infinite M200 plate reader (Tecan) and normalized to the positive control cells.

Hemolysis assay

Red blood cells (RBCs) were washed three times in PBS by centrifuging at 4 °C and 3,000 x g for 5 min and diluted to 5% in PBS. The desired peptide concentrations diluted in PBS were added into a 96-well plate. Next, 5% RBCs in PBS were added into the wells and incubated for 24 h at 37 °C and 5% CO_2. As a positive control, RBCs were mixed with 10% Triton X-100 in PBS. After the incubation time was completed, the plate was centrifuged at 2,500 x g for 3 min at RT and 100 µL of the supernatant was transferred into a fresh 96-well plate. Lastly, the absorption (λ=560 nm) was measured on the infinite M200 plate reader (Tecan). For the evaluation, the measured absorption of the Triton-X-100 treatment was set to a hemolysis rate of 100%.

Time-lapse Imaging

30,000 HeLa cells were seeded into an 8-well. Cells were stained for 10 min with the Hoechst 33342 nuclear dye and transferred to the microscope UltraView VoX Spinning Disk Confocal Microscope (Perkin Elmer). 5 µM CF-DC-2 were added to the cells. Cells were imaged on a Plan-Apo Tirf 60X oil objective (NA 1.49, Nikon) at 37°C and 5% CO_2. The first image was taken immediately, followed by an image every 10 sec for 30 min in total. The video was generated using Fiji.

Peptide incubation in media – L-cystine and identification of peptide species

95,000 HeLa cells were seeded in a 24-well plate. The next day, 5 µM CF-DC-2 was diluted in either serum-free medium or serum-free medium lacking L-cystine and L-methionine. Before addition to the cells, 50 µL of the peptide solution was then transferred to a reaction vial and

mixed with 5 µl TFA. Cells were incubated with the peptide solution for 30 min at 37 °C and 5% CO_2. After this incubation time, again 50 µL of the supernatant was removed and mixed with 5 µL TFA. C18 ZipTips were used to desalt the samples and the eluate in 50% ACN in ddH_2O + 0.1% FA was diluted 1:1 with 10% ACN in ddH_2O + 0.1% FA. Finally, the different peptide species were analyzed by analytical HPLC ESI-MS (LTQ XL, Thermo Scientific) using a gradient of 10% to 60% of acetonitrile (ACN) in ddH_2O + 0.1% formic acid (FA) on an Aeris 3.6 µm PEPTIDE XB-C18 100 LC column (Phenomenex).

Supplementary Figures

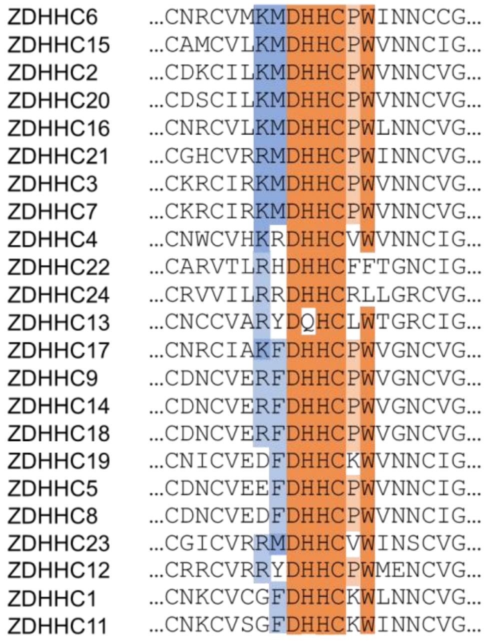

Fig. S1: Multiple Sequence Alignment performed with the Clustal Omega Tool from EMBL-EBI comparing all human ZDHHC enzymes [71].

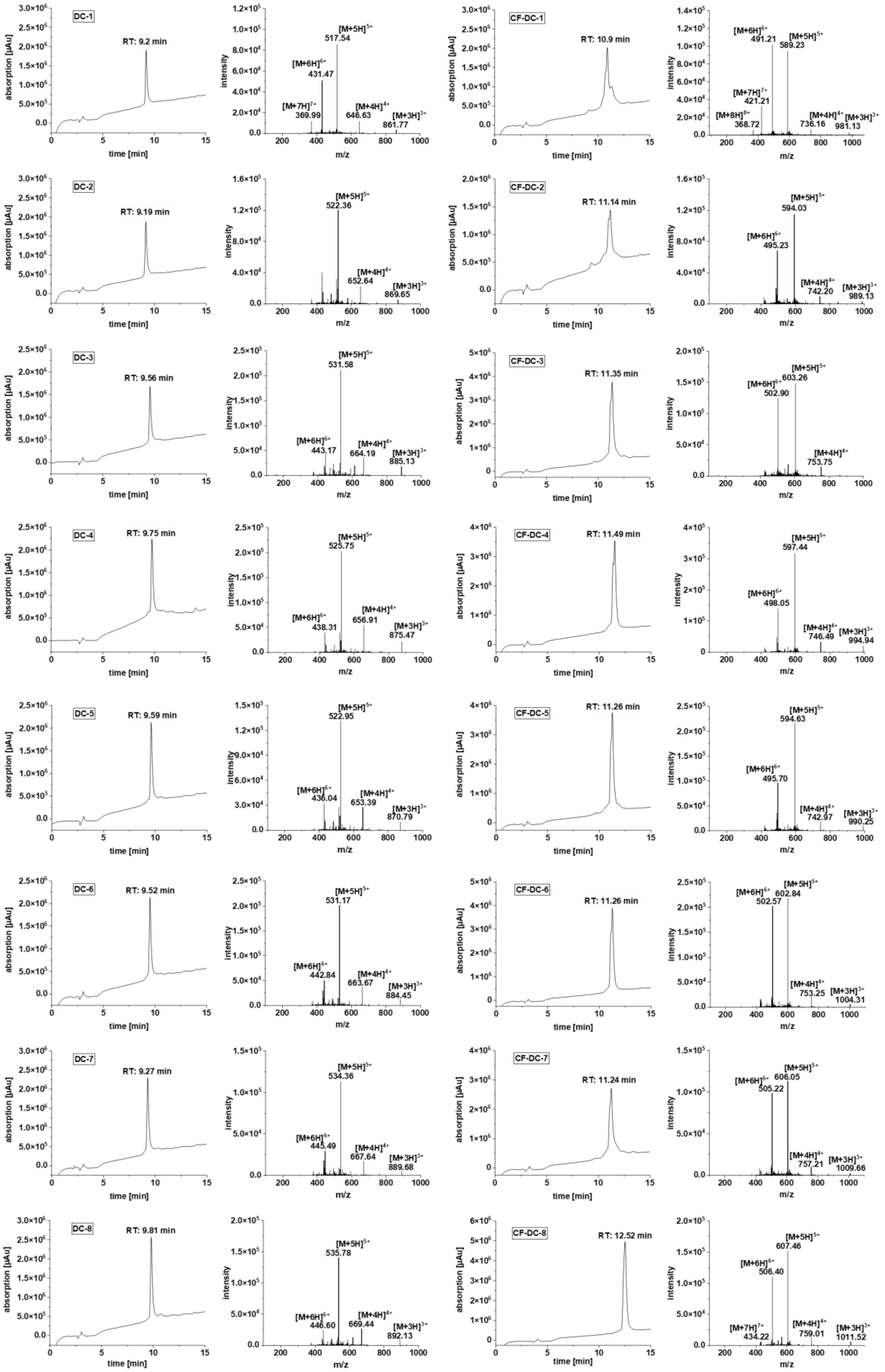
DC-1
RT: 9.2 min
[M+5H]5+ 517.54
[M+6H]6+ 431.47
[M+7H]7+ 369.99
[M+4H]4+ 646.63
[M+3H]3+ 861.77
CF-DC-1
RT: 10.9 min
[M+6H]6+ 491.21
[M+5H]5+ 589.23
[M+7H]7+ 421.21
[M+8H]8+ 368.72
[M+4H]4+ 736.16
[M+3H]3+ 981.13
DC-2
RT: 9.19 min
[M+5H]5+ 522.36
[M+4H]4+ 652.64
[M+3H]3+ 869.65
CF-DC-2
RT: 11.14 min
[M+5H]5+ 594.03
[M+6H]6+ 495.23
[M+4H]4+ 742.20
[M+3H]3+ 989.13
DC-3
RT: 9.56 min
[M+5H]5+ 531.58
[M+6H]6+ 443.17
[M+4H]4+ 664.19
[M+3H]3+ 885.13
CF-DC-3
RT: 11.35 min
[M+5H]5+ 603.26
[M+6H]6+ 502.90
[M+4H]4+ 753.75
DC-4
RT: 9.75 min
[M+5H]5+ 525.75
[M+6H]6+ 438.31
[M+4H]4+ 656.91
[M+3H]3+ 875.47
CF-DC-4
RT: 11.49 min
[M+5H]5+ 597.44
[M+6H]6+ 498.05
[M+4H]4+ 746.49
[M+3H]3+ 994.94
DC-5
RT: 9.59 min
[M+5H]5+ 522.95
[M+6H]6+ 436.04
[M+4H]4+ 653.39
[M+3H]3+ 870.79
CF-DC-5
RT: 11.26 min
[M+5H]5+ 594.63
[M+6H]6+ 495.70
[M+4H]4+ 742.97
[M+3H]3+ 990.25
DC-6
RT: 9.52 min
[M+5H]5+ 531.17
[M+6H]6+ 442.84
[M+4H]4+ 663.67
[M+3H]3+ 884.45
CF-DC-6
RT: 11.26 min
[M+6H]6+ 502.57
[M+5H]5+ 602.84
[M+4H]4+ 753.25
[M+3H]3+ 1004.31
DC-7
RT: 9.27 min
[M+5H]5+ 534.36
[M+6H]6+ 445.49
[M+4H]4+ 667.64
[M+3H]3+ 889.68
CF-DC-7
RT: 11.24 min
[M+6H]6+ 505.22
[M+5H]5+ 606.05
[M+4H]4+ 757.21
[M+3H]3+ 1009.66
DC-8
RT: 9.81 min
[M+5H]5+ 535.78
[M+6H]6+ 446.60
[M+4H]4+ 669.44
[M+3H]3+ 892.13
CF-DC-8
RT: 12.52 min
[M+5H]5+ 607.46
[M+6H]6+ 506.40
[M+7H]7+ 434.22
[M+4H]4+ 759.01
[M+3H]3+ 1011.52
absorption [µAu]
time [min]
intensity
m/z

Fig. S2: LC-MS analysis of unlabeled and CF-labeled DC-1-DC-8. The UV chromatograms were recorded using a linear gradient of 10-60% ACN in ddH_2O +0.1% TFA in 15 min. Some CF-labelled variants show double peaks, which originate from the isomers of 5(6)-carboxyfluorescein. The mass spectra demonstrate the m/z signals that correspond to the quasi-molecular ions of the respective peak of the corresponding peptide.

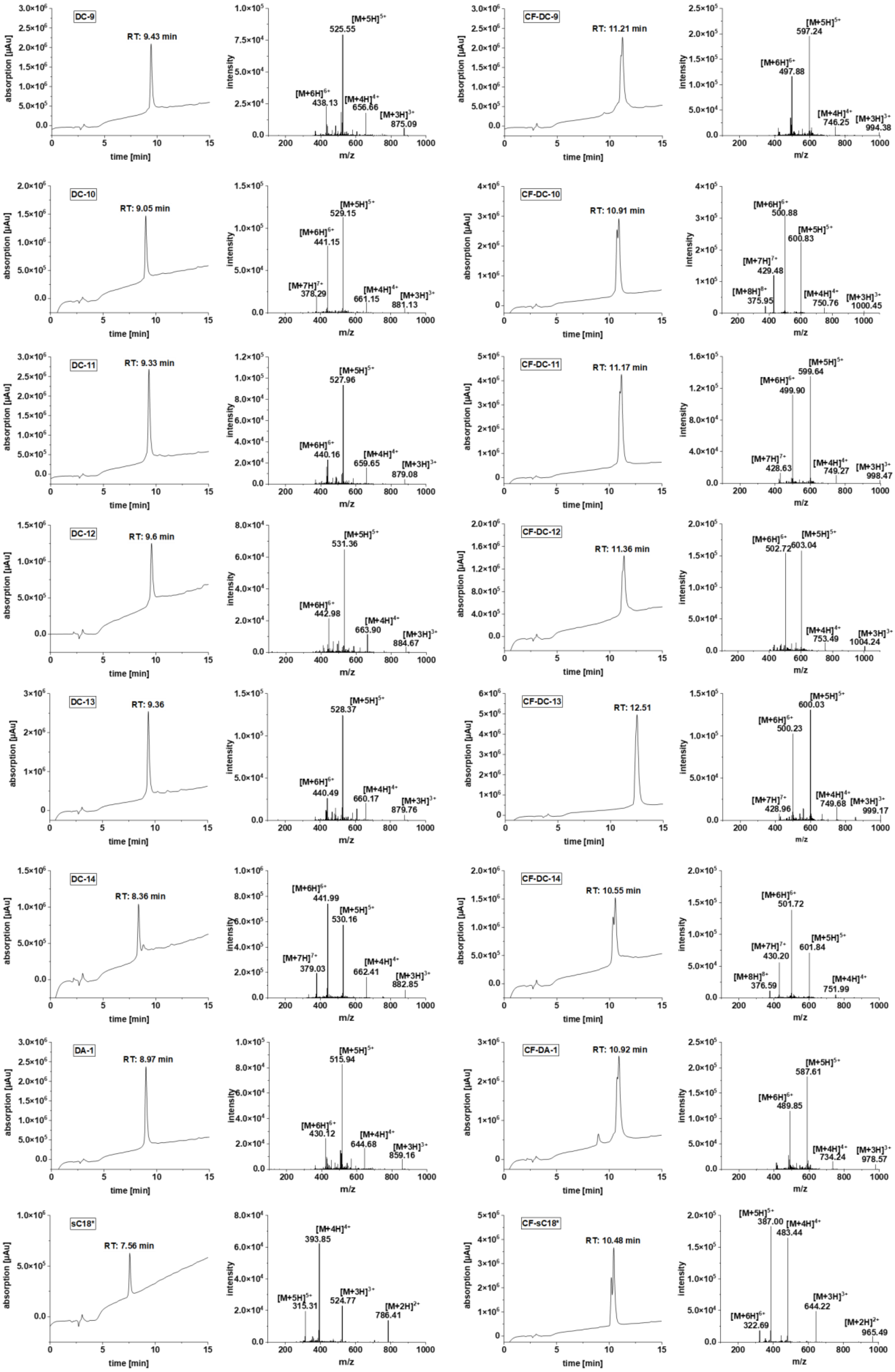

DC-9
RT: 9.43 min
[M+5H]5+ 525.55
[M+6H]6+ 438.13
[M+4H]4+ 656.66
[M+3H]3+ 875.09
CF-DC-9
RT: 11.21 min
[M+5H]5+ 597.24
[M+6H]6+ 497.88
[M+4H]4+ 746.25
[M+3H]3+ 994.38
DC-10
RT: 9.05 min
[M+5H]5+ 529.15
[M+6H]6+ 441.15
[M+7H]7+ 378.29
[M+4H]4+ 661.15
[M+3H]3+ 881.13
CF-DC-10
RT: 10.91 min
[M+6H]6+ 500.88
[M+5H]5+ 600.83
[M+7H]7+ 429.48
[M+8H]8+ 375.95
[M+4H]4+ 750.76
[M+3H]3+ 1000.45
DC-11
RT: 9.33 min
[M+5H]5+ 527.96
[M+6H]6+ 440.16
[M+4H]4+ 659.65
[M+3H]3+ 879.08
CF-DC-11
RT: 11.17 min
[M+5H]5+ 599.64
[M+6H]6+ 499.90
[M+7H]7+ 428.63
[M+4H]4+ 749.27
[M+3H]3+ 998.47
DC-12
RT: 9.6 min
[M+5H]5+ 531.36
[M+6H]6+ 442.98
[M+4H]4+ 663.90
[M+3H]3+ 884.67
CF-DC-12
RT: 11.36 min
[M+6H]6+ 502.72
[M+5H]5+ 603.04
[M+4H]4+ 753.49
[M+3H]3+ 1004.24
DC-13
RT: 9.36
[M+5H]5+ 528.37
[M+6H]6+ 440.49
[M+4H]4+ 660.17
[M+3H]3+ 879.76
CF-DC-13
RT: 12.51
[M+5H]5+ 600.03
[M+6H]6+ 500.23
[M+7H]7+ 428.96
[M+4H]4+ 749.68
[M+3H]3+ 999.17
DC-14
RT: 8.36 min
[M+6H]6+ 441.99
[M+5H]5+ 530.16
[M+7H]7+ 379.03
[M+4H]4+ 662.41
[M+3H]3+ 882.85
CF-DC-14
RT: 10.55 min
[M+6H]6+ 501.72
[M+7H]7+ 430.20
[M+5H]5+ 601.84
[M+8H]8+ 376.59
[M+4H]4+ 751.99
DA-1
RT: 8.97 min
[M+5H]5+ 515.94
[M+6H]6+ 430.12
[M+4H]4+ 644.68
[M+3H]3+ 859.16
CF-DA-1
RT: 10.92 min
[M+5H]5+ 587.61
[M+6H]6+ 489.85
[M+4H]4+ 734.24
[M+3H]3+ 978.57
sC18*
RT: 7.56 min
[M+4H]4+ 393.85
[M+5H]5+ 315.31
[M+3H]3+ 524.77
[M+2H]2+ 786.41
CF-sC18*
RT: 10.48 min
[M+5H]5+ 387.00
[M+4H]4+ 483.44
[M+3H]3+ 644.22
[M+6H]6+ 322.69
[M+2H]2+ 965.49
absorption [µAu]
time [min]
intensity
m/z

Fig. S3: LC-MS analysis of unlabelled and CF-labelled DC-9-DC14, DA-1 and sC18*. The UV chromatograms were recorded using a linear gradient of 10-60% ACN in ddH_2O +0.1% TFA in 15 min. Some CF-labelled variants show double peaks, which originate from the isomers of 5(6)-carboxyfluorescein. The mass spectra demonstrate the m/z signals that correspond to the quasi-molecular ions of the respective peak of the corresponding peptide.

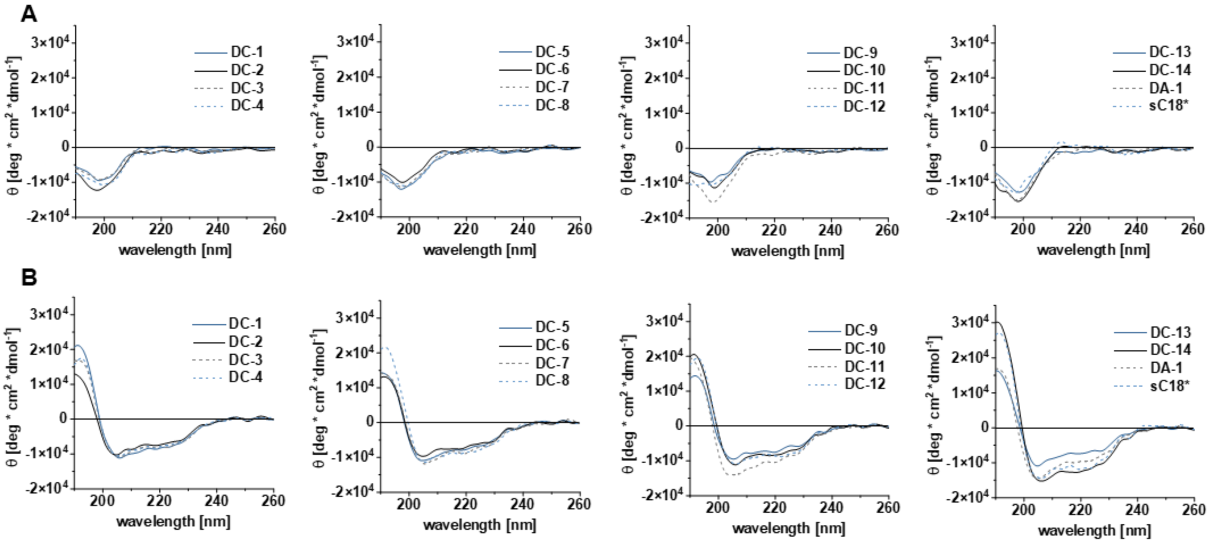

Fig. S4: CD spectra of all DC peptides in (A) 10 mM phosphate buffer, pH 7.0 or (B) 10 mM phosphate buffer, pH 7.0 supplemented with 50% (v/v) trifluoroethanol (TFE).

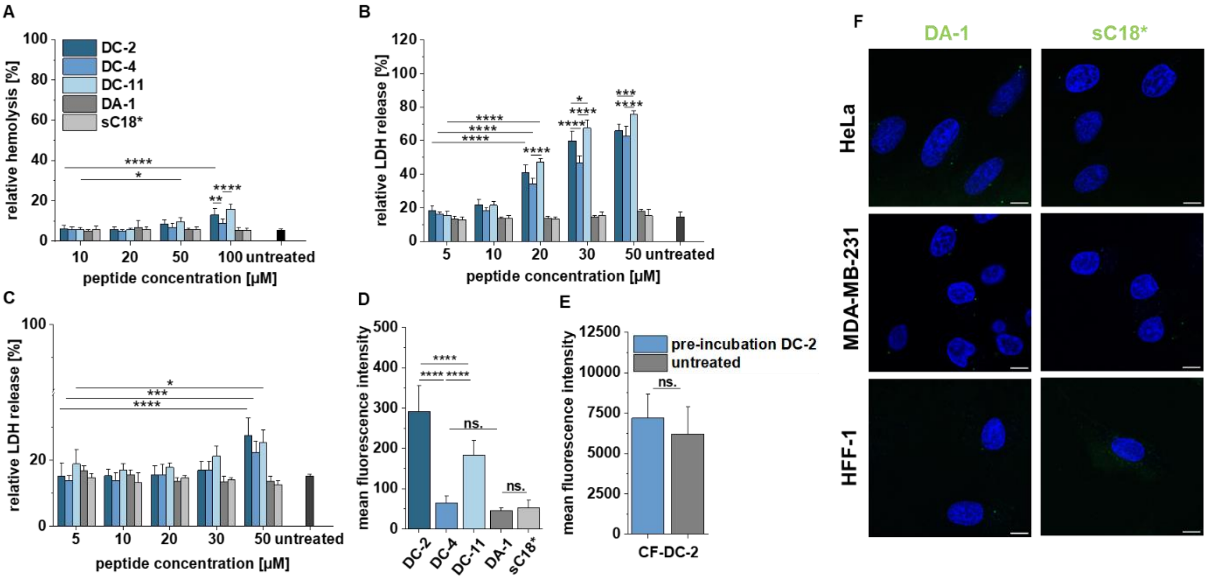

Fig. S5: (A) Hemolysis assay using red blood cells treated for 24 h with peptides diluted in PBS (n=3). A positive control was treated with Triton-X-100 and set to 100% hemolysis rate. **(B)** Lactate dehydrogenase (LDH) release assay: HeLa cells were treated for 1 h with CF-labeled peptides (n=2). **(C)** LDH release assay: HeLa cells were treated for 1 h with CF-labeled peptide. The supernatant was removed and cells were further incubated for 5 h in serum-free medium (n=2). A control treated with lysis solution was set to 100% LDH release. Statistical analyses were performed using a two-way ANOVA (*P ≤ 0.05, **P ≤ 0.01, ***P ≤ 0.001, ****P ≤ 0.0001). **(D)** Cellular uptake in HeLa cells quantified by using flow cytometry. Cells were incubated for 30 min with 1 µM CF-labeled peptides (n=4). Statistical analysis was performed using a one-way ANOVA (*P ≤ 0.05, **P ≤ 0.01, ***P ≤ 0.001, ****P ≤ 0.0001, ns: not significant). **(E)** HeLa cells were pre-treated with 5 µM DC-2 for 30 min or kept untreated, followed by treatment for 30 min with 5 µM CF-DC-2 (n=3). Statistical analysis was performed using an unpaired T-test (****P ≤ 0.0001, ns: not significant). **(F)** Cellular uptake studies of control peptides into HeLa, MDA-MB-231 and HFF-1 cells using live cell fluorescence microscopy. Cells were incubated for 30 min with 5 µM CF-labeled peptides. Blue: Hoechst 33342 nuclear stain, Green: CF-labeled peptide, Scale Bar: 10 µm.

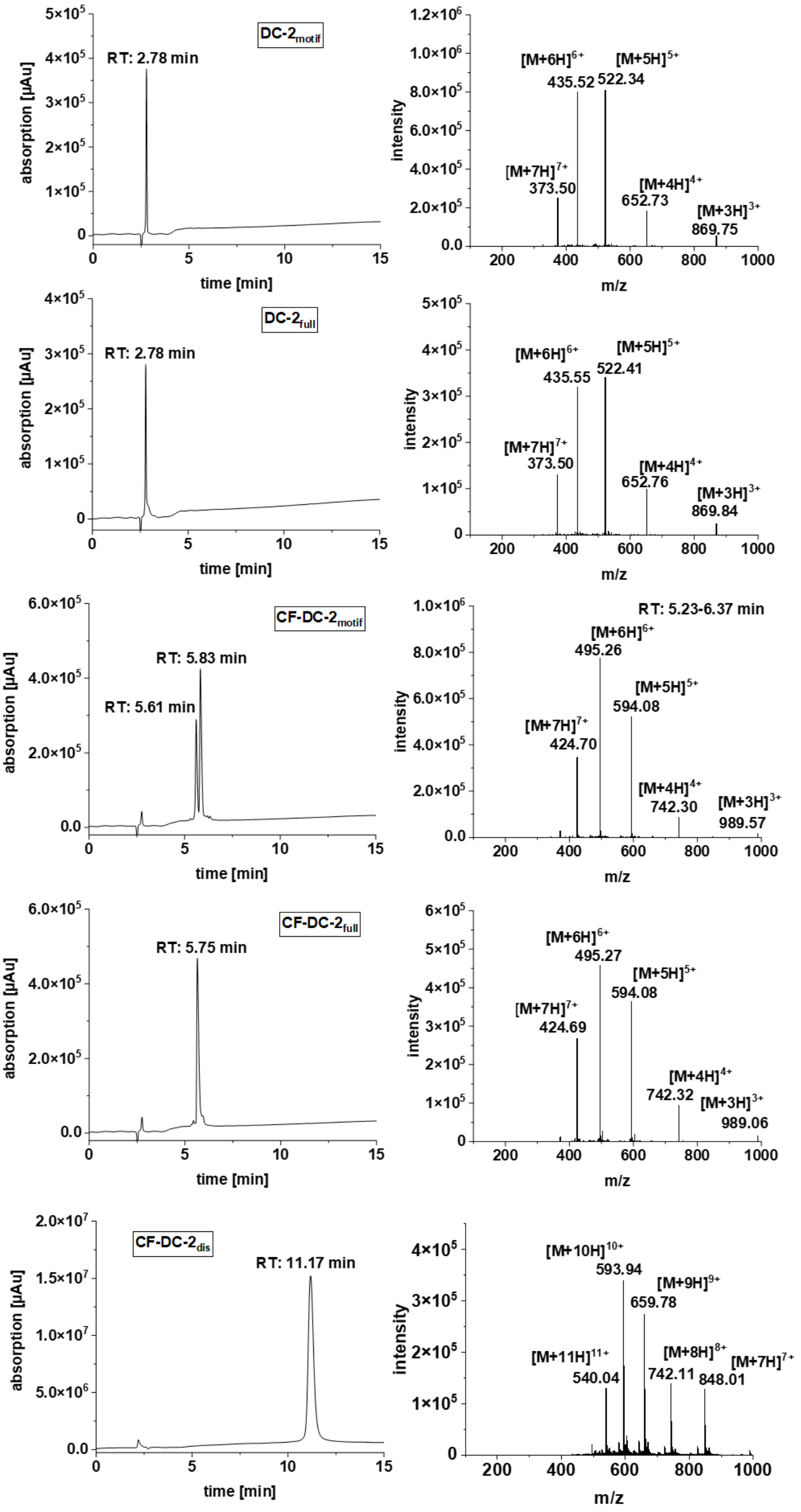

Fig. S6: LC-MS analysis of purified unlabelled and CF-labelled DC-2$_{motif}$, DC-2$_{full}$ and CF-DC-2$_{dis}$. The UV chromatograms were recorded using a linear gradient of 10-60% ACN in ddH_2O +0.1% FA/TFA in 15 min. CF-labelled DC-2$_{motif}$ shows a double peak that originates from the isomers of 5(6)-carboxyfluorescein. The mass spectra demonstrate the m/z signals that correspond to the quasi-molecular ions of the respective peak of the corresponding peptide.

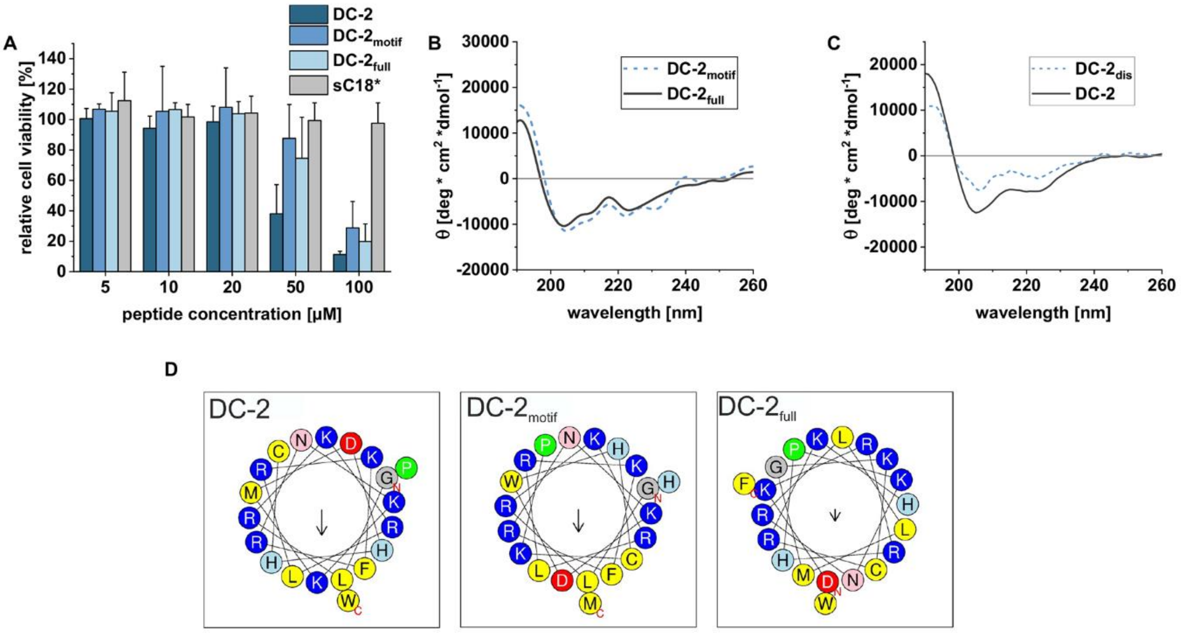

Fig. S7: (A) HeLa cells were incubated for 24 h with different µM concentrations of peptides (n=4). An untreated control was set to 100% cellular viability. CD spectra of **(B)** scrambled DC peptides or **(C)** CF-DC-2 and CF-DC-2$_{dis}$ in 10 mM phosphate buffer, pH 7.0 supplemented with 50% (v/v) trifluoroethanol (TFE). **(D)** Helical wheel projections generated with Heliquest [72]. Color code: Blue, light blue, red, and light red show polar residues. Yellow, grey, and green shown non-polar residues. The arrow indicates the hydrophobic moment.

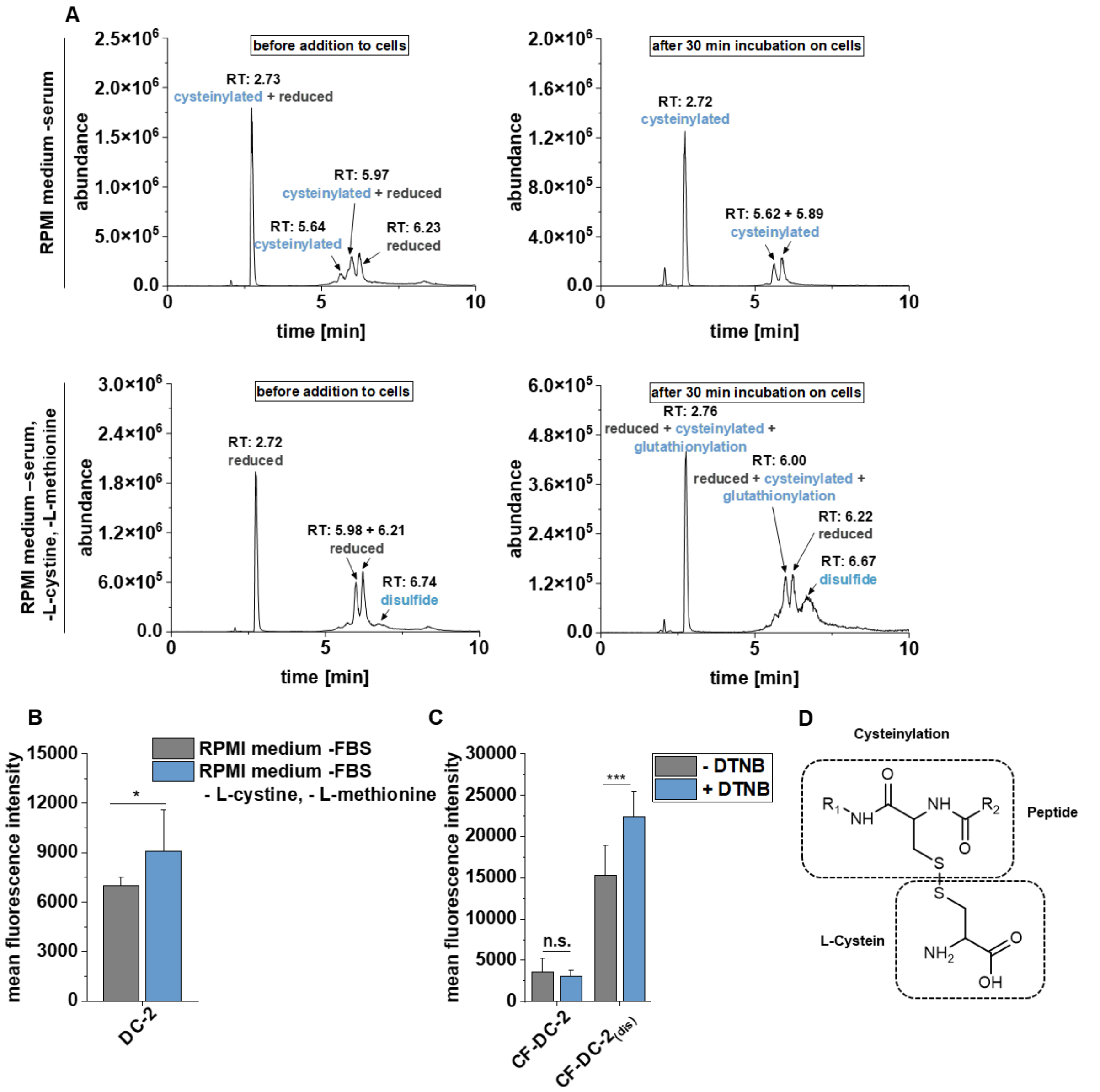

Fig. S8: (A) LC-MS analysis of CF-DC-2 incubated in serum-free medium or serum-free medium -L-cystine, -L-methionine. **(B)** Flow cytometry analysis of HeLa cells incubated for 30 min with 5 µM CF-DC-2 incubated in different media conditions (n=3). Statistical analysis was performed using an unpaired T-test (*P ≤ 0.05). **(C)** Flow cytometry analysis of HeLa cell pre-treated for 5 min with 1.2 mM DTNB followed by incubation with 1 µM peptide and 1 mM DTNB for 30 min (n=3). Statistical analysis was performed using an unpaired T-test (***P ≤ 0.001). **(D)** S-cysteinylation of peptide.

DC-2

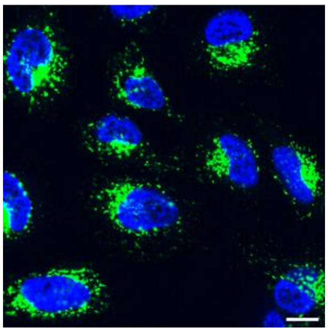

Fig. S9: Analysis of peptide uptake into HeLa cells using live cell fluorescence microscopy. Cells were incubated for 6 h with 5 µM CF-labeled DC-2. Blue: Hoechst 33342 nuclear stain, Green: CF-labeled peptide, Scale Bar: 10 µm.

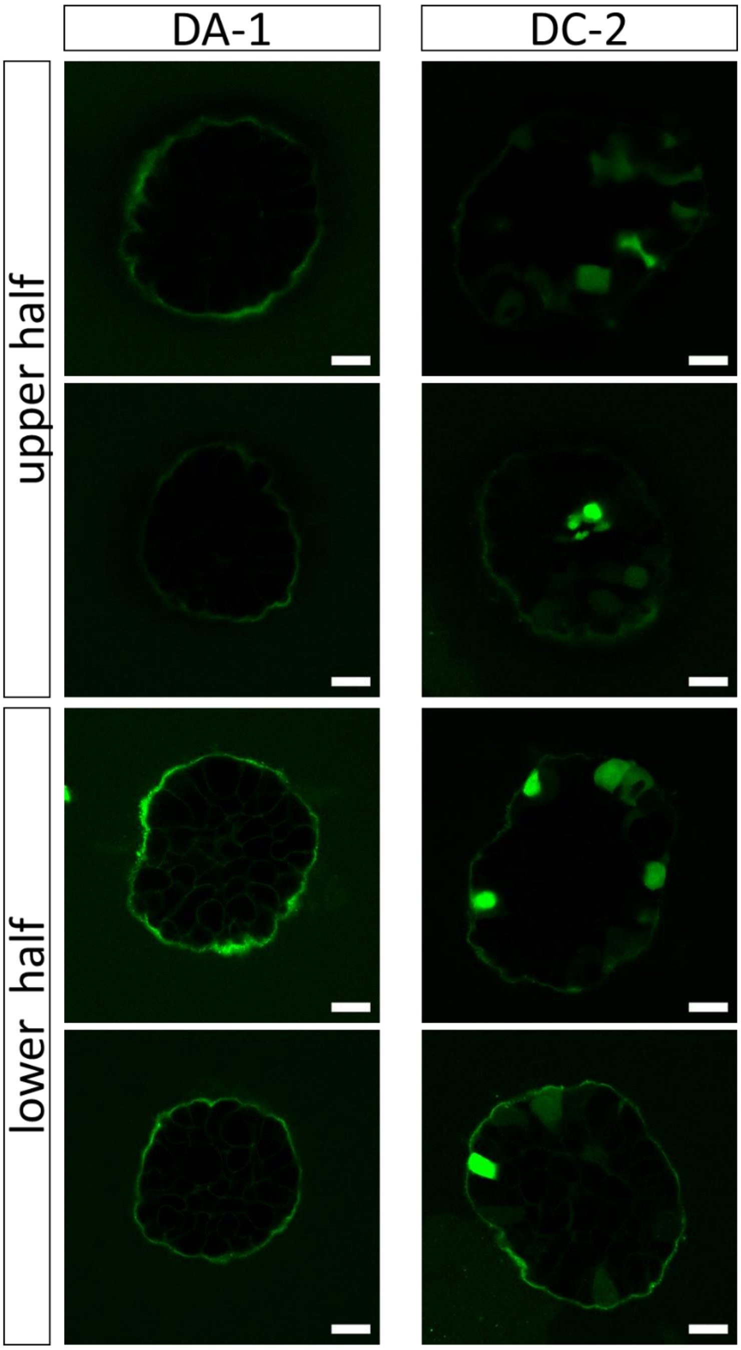

Fig. S10: Confocal imaging of MCF10A breast acini imaged at different microscopic image planes (z-planes). 10-days-old acini were treated with 10 µM CF-DC-2 or CF-DA-1 for 25 min. Green: CF-labeled peptide scale bar: 20 µm. The experiment was performed n≤2 with one n including 6 acini.

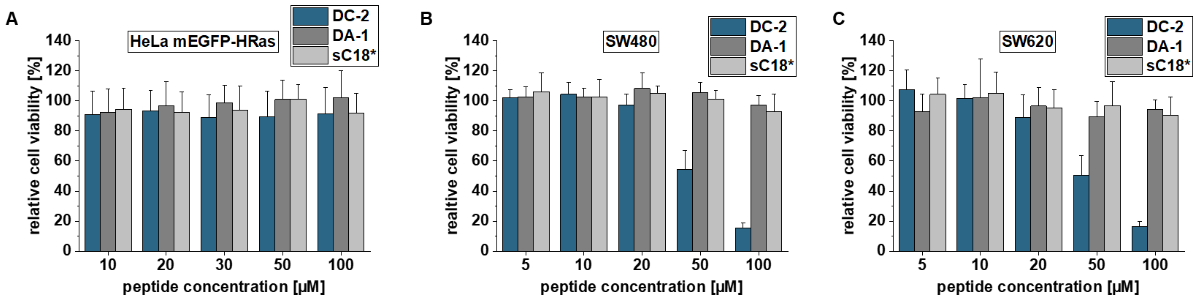

Fig. S11. Cell viability assays. **(A)** HeLa mEGFP-HRas, **(B)** SW480, and **(C)** SW620 cells were incubated for 24 h with different concentrations of the peptides DC-2, DA-1 and sC18* (n=3). An untreated control was set to 100% cellular viability.

Video 1: Confocal time-lapse analysis of peptide uptake into HeLa cells. Cells were incubated with 5 µM CF-labeled DC-2 and imaged every 10 sec for a total of 30 min. Blue: Hoechst 33342 nuclear stain, Green: CF-labeled peptide, Scale Bar: 10 µm.

Tab. ST1: List of all unlabeled peptides synthesized within this work. Listed are the sequence, calculated (calc.) and experimentally (exp.) determined molecular weights (MW), purities and net charge at pH 7.0. All peptides are *C*-terminally amidated**.** (grey: sC18*; blue: DHHC motif)

Name	Sequence	$MW_{(calc.)}$ [Da]	$MW_{(exp.)}$ [Da]	Purity [%]	Net Charge
**DC-1**	GLRKRLRKFRNKGFDHHCKW-NH_2	2582.06	2582.86	93	+8
**DC-2**	GLRKRLRKFRNKKMDHHCPW-NH_2	2606.15	2606.44	93	+8
**DC-3**	GLRKRLRKFRNKRFDHHCVW-NH_2	2652.16	2652.77	92	+8
**DC-4**	GLRKRLRKFRNKEFDHHCPW-NH_2	2623.07	2623.67	93	+6
**DC-5**	GLRKRLRKFRNKDFDHHCPW-NH_2	2609.04	2609.73	94	+6
**DC-6**	GLRKRLRKFRNKRFDHHCPW-NH_2	2650.14	2650.73	96	+8
**DC-7**	GLRKRLRKFRNKRYDHHCPW-NH_2	2666.14	2666.59	92	+8
**DC-8**	GLRKRLRKFRNKRYDQHCLW-NH_2	2673.17	2673.66	94	+8
**DC-9**	GLRKRLRKFRNKKFDHHCPW-NH_2	2622.13	2622.61	96	+8
**DC-10**	GLRKRLRKFRNKDFDHHCKW-NH_2	2640.10	2640.73	92	+7
**DC-11**	GLRKRLRKFRNKRMDHHCPW-NH_2	2634.16	2634.65	95	+8
**DC-12**	GLRKRLRKFRNKRHDHHCFF-NH_2	2651.13	2651.57	94	+8
**DC-13**	GLRKRLRKFRNKRMDHHCVW-NH_2	2636.18	2636.69	93	+8
**DC-14**	GLRKRLRKFRNKRRDHHCRL-NH_2	2645.17	2645.83	98	+10
**DA-1**	GLRKRLRKFRNKKMDHHAPW-NH_2	2574.08	2574.67	97	+8
**sC18***	GLRKRLRKFRNK-NH_2	1570.94	1571.52	98	+8

Tab. ST2: List of all CF-labeled peptides synthesized within this work. Listed are the sequence, calculated (calc.) and experimentally (exp.) determined molecular weights (MW), purities and net charge at pH 7.0. All peptides are *C*-terminally amidated. (grey: sC18*; blue: DHHC motif)

Name	Sequence	$MW_{(calc.)}$ [Da]	$MW_{(exp.)}$ [Da]	Purity [%]	Net Charge
**CF-DC-1**	CF-GLRKRLRKFRNKGFDHHCKW-NH_2	2940.38	2941.11	87	+7
**CF-DC-2**	CF-GLRKRLRKFRNKKMDHHCPW-NH_2	2964.47	2964.93	87	+7
**CF-DC-3**	CF-GLRKRLRKFRNKRFDHHCVW-NH_2	3010.48	3011.23	96	+7
**CF-DC-4**	CF-GLRKRLRKFRNKEFDHHCPW-NH_2	2981.39	2982.07	95	+5
**CF-DC-5**	CF-GLRKRLRKFRNKDFDHHCPW-NH_2	2967.36	2968.00	93	+5
**CF-DC-6**	CF-GLRKRLRKFRNKRFDHHCPW-NH_2	3008.46	3009.39	96	+7
**CF-DC-7**	CF-GLRKRLRKFRNKRYDHHCPW-NH_2	3024.46	3025.35	91	+7
**CF-DC-8**	CF-GLRKRLRKFRNKRYDQHCLW-NH_2	3031.49	3032.12	98	+7
**CF-DC-9**	CF-GLRKRLRKFRNKKFDHHCPW-NH_2	2980.45	2980.91	91	+7
**CF-DC-10**	CF-GLRKRLRKFRNKDFDHHCKW-NH_2	2998.42	2999.13	98	+6
**CF-DC-11**	CF-GLRKRLRKFRNKRMDHHCPW-NH_2	2992.48	2993.10	98	+7
**CF-DC-12**	CF-GLRKRLRKFRNKRHDHHCFF-NH_2	3009.45	3010.05	98	+7
**CF-DC-13**	CF-GLRKRLRKFRNKRMDHHCVW-NH_2	2994.50	2995.10	95	+7
**CF-DC-14**	CF-GLRKRLRKFRNKRRDHHCRL-NH_2	3003.49	3004.32	96	+9
**CF-DA-1**	CF-GLRKRLRKFRNKKMDHHAPW-NH_2	2932.40	2932.96	87	+7
**CF-sC18***	CF-GLRKRLRKFRNK-NH_2	1929.26	1929.71	98	+7

References

[71] F. Sievers, D. G. Higgins, Methods Mol. Biol. 2021, 2231, 3.

[72] R. Gautier, D. Douguet, B. Antonny, G. Drin, Bioinformatics 2008, 24, 2101.
